# A kidney-protective mechanism via cellular oxidative stress reduction induced by CD5L protein

**DOI:** 10.1038/s41420-026-03171-2

**Published:** 2026-05-23

**Authors:** Kai Kudo, Takashi Ikeda, Kazutaka Ikeda, Natsumi Maehara, Aika Hirota, Yuri Yoshikawa, Haruka Mori, Masumi Takayama, Keisuke Yasuda, Tetsushi Tezuka, Toshio Takagi, Satoko Arai, Toru Miyazaki

**Affiliations:** 1 The Institute for AIM Medicine (IAM), Tokyo, Japan; 2 Department of Urology, Tokyo Women’s Medical University, Tokyo, Japan; 3 Department of Applied Genomics, Laboratory of Biomolecule Analysis, Kazusa DNA Research Institute, Chiba, Japan; 4 LEAP, Japan Agency for Medical Research and Development, Tokyo, Japan; 5 Laboratoire d’ImmunoRhumatologie Moléculaire, Plateforme GENOMAX, Institut National de la Santé et de la Recherche Médicale UMR_S 1109, Faculté de Médecine, Fédération Hospitalo-Universitaire OMICARE, Fédération de Médecine Translationnelle de Strasbourg, Laboratory of Excellence TRANSPLANTEX, Université de Strasbourg, Strasbourg, France; 6 Present Address: Laboratory of Food Chemistry, Department of Applied Biological Chemistry, Graduate School of Agricultural and Life Sciences, The University of Tokyo, Tokyo, Japan

**Keywords:** Lipids, Kidney diseases

## Abstract

Chronic kidney disease (CKD) encompasses multiple pathogenic mechanisms manifested by inflammation, fibrosis, oxidative stress and cell death. We previously showed that circulating protein CD5L (or AIM) ameliorates acute kidney injury, so we explored its effect in a mouse model of unilateral ureteral obstruction (UUO)-induced renal fibrosis. Here, we show a unique kidney-protective pathway mediated by CD5L. CD5L is endocytosed into renal epithelial cells, where it reduces oxidative stress, decreasing cell injury and death. This effect is supported by both a cysteine-dependent direct antioxidant activity of CD5L and enhancement of Nrf2-associated antioxidant responses. In addition, our data suggest that suppression of sphingomyelinase activity and reduction of cellular ceramide may contribute, at least in part, to CD5L-associated augmentation of Nrf2 nuclear transport. These effects depend on the reactive cysteine residue on the CD5L surface. Consistent with these findings, recombinant CD5L treatment in UUO mice reduces sphingomyelinase activity, activates Nrf2, and lowers oxidative stress, alleviating inflammation, fibrosis, and kidney injury. Our findings uncover a novel antioxidant pathway mediated by CD5L with potential implications for CKD-associated fibrotic mechanisms.

## Introduction

Chronic kidney disease (CKD) results from progressive and permanent damage to the kidney, and it can be caused by a variety of conditions, such as diabetes and hypertension. It affects approximately 10% of the global population, and its prevalence has increased markedly over recent decades in parallel with population ageing and the rising incidence of diabetes, hypertension and obesity [1–5]. Although CKD arises from diverse etiologies, its progression is driven by multiple pathogenic mechanisms, including oxidative stress, persistent inflammation, tubulointerstitial fibrosis, microvascular injury, and progressive nephron loss [6–15]. Because these mechanisms reinforce one another, CKD often advances through a vicious cycle that irreversibly exacerbates the disease.

As renal function declines, metabolites and organic toxins accumulate in the circulation, many of which can further promote oxidative stress and exacerbate tissue injury [16–21]. Tremendous effort has therefore been devoted to developing therapies that slow CKD progression, including strategies targeting renal fibrosis [1, 22]. Some currently available strategies have been shown to delay or slow disease progression to a certain degree during relatively early stages of the disease [23–28]. Nevertheless, CKD often continues to progress in a substantial proportion of patients, indicating persistent residual risk and the need to identify additional kidney-protective mechanisms. Antioxidant-based approaches, including pharmacologic activation of nuclear factor erythroid 2-related factor 2 (Nrf2), have also been investigated, but their clinical outcomes remain controversial, and their translational utility remains uncertain [29–33]. This may partly reflect the fact that ROS also serve essential signaling functions. Accordingly, cellular redox homeostasis must be tightly maintained through a balance between ROS production and elimination so that normal cell function is preserved without tipping cells toward either oxidative or reductive stress [34–38]. These considerations highlight the need to identify endogenous protective mechanisms that can attenuate oxidative stress in a more physiologically integrated manner.

CD5L-like protein (CD5L, also called apoptosis inhibitor of macrophage; AIM) is a circulating protein produced by tissue macrophages, present in the blood at relatively high levels (~5 μg/mL in humans; ~3 μg/mL in mice). CD5L consists of three scavenger receptor cysteine rich (SRCR) domains, and the second domain (SRCR2 domain) harbors a unique solitary cysteine residue on the protein surface [39, 40]. We initially identified CD5L as a supporter of macrophage survival, but it is now also known as a facilitator of repair in many diseases through the promotion of phagocytic removal of a wide range of extracellular organic wastes, including dead cell debris and damage-associated molecular patterns [41–50]. This scavenger-like function depends on the reactive cysteine in the SRCR2 domain, which enables CD5L to bind target materials through disulfide bond formation [41, 43, 45]. Interestingly, alongside the binding to wastes in the extracellular environment, CD5L is endocytosed into various cell types through scavenger receptors and functions within the cytosol [49, 51, 52]. Under physiological conditions, CD5L is largely associated with the IgM pentamer in the serum [53, 54], whereas in disease states, including kidney disease, it can dissociate from IgM, thereby exposing its cysteine residue in a reactive form [41, 55]. This suggests that in disease conditions, CD5L may serve as an endogenous protective factor that helps counter further oxidative and inflammatory injury.

As our group and others have shown that CD5L can ameliorate acute kidney injury (AKI) [43, 56–58], we investigated its effect in UUO-induced kidney injury, anticipating that CD5L might interrupt tissue injury by promoting removal of inflammatory organic wastes. During this assessment, we identified previously unrecognized antioxidant-associated mechanisms underlying the kidney-protective effect of CD5L. Our findings suggested that, after cellular uptake, CD5L attenuates oxidative stress through a cysteine-dependent antioxidant effect together with enhancement of Nrf2-associated responses, potentially involving modulation of ceramide-related lipid signaling.

## Results

### Manifold kidney-protective pathways activated by CD5L distinguished in the UUO mouse model

To test the potential impacts of CD5L in fibrotic kidney injury, we utilized the unilateral ureteral obstruction (UUO) model, a well-established method for inducing accelerated renal fibrosis in mice [59]. Wild-type (WT) and CD5L-deficient (CD5L KO) mice on C57BL/6J (B6) strain were subjected to UUO and received daily intraperitoneal injections of rCD5L (0.5 mg/mouse) or an equal volume of PBS (as the control). The obstructed kidneys were analyzed on day 12 (Supplementary Fig. 1A). Immunofluorescence analysis demonstrated detectable CD5L protein within renal tubular regions, including LRP2-positive structures, after exogenous rCD5L administration, with endogenous CD5L signal also observed in WT kidneys (Supplementary Fig. 1B). Notably, since the contralateral kidney remained intact, the mice did not develop renal failure, and serum biomarkers for renal function remained within normal ranges throughout the experiment.

**Fig. 1:**
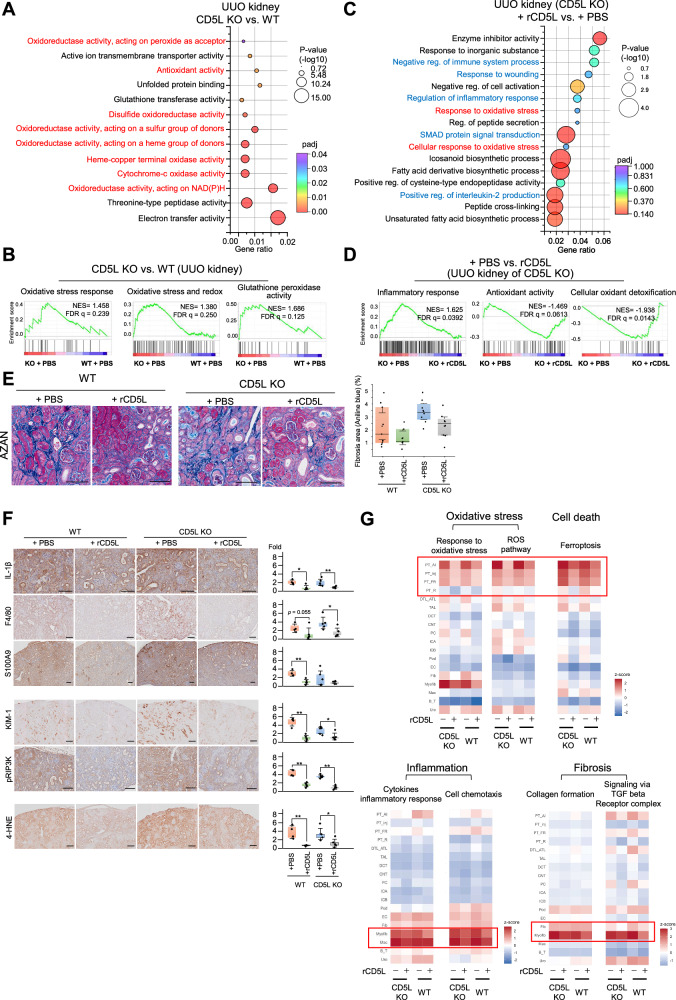
CD5L modulates oxidative stress in the kidney and ameliorates UUO-induced tissue damage. **A** GO enrichment analysis of RNA-seq results from UUO kidneys of CD5L KO and WT mice injected with PBS. Representative significantly altered Molecular Function terms are shown; oxidative stress-related terms are highlighted in red. The gene ratio (x-axis) represents the percentage of differentially expressed genes (DEGs) associated with a given GO term. Circle size indicates the p-value (-log10), while circle color represents the adjusted p-value based on the provided color palette. **B** GSEA of UUO kidneys of PBS-injected CD5L KO and WT mice. False discovery rate (FDR) q-value and Enrichment Score (ES) are shown in the figure. Results of the pathways related to oxidative stress are presented. **C** GO enrichment analysis of RNA-seq results from CD5L KO mice injected with PBS or rCD5L. Representative ontologies related to oxidative stress (red) and inflammation (blue) are shown. **D** GSEA of UUO kidneys in CD5L KO mice injected with either PBS- or rCD5L. Results of the pathways related to oxidative stress and inflammation are presented. **E** AZAN staining of UUO kidneys. Fibrotic area (blue-stained area) in the cortex was quantified as the percentage of Aniline Blue-positive area. Scale bar, 50 µm. **F** Immunohistochemistry for different antigens performed in operated kidneys. Representative photos are shown. Quantification of DAB-positive areas in UUO kidney tissues used for (**E**). Scale bar, 100 µm. The positivity was calculated as the percentage relative to the nuclear area on the same section and presented as relative values to results in WT+rCD5L group. **G** Heatmaps showing z-scored median pathway activity across major renal cell populations in UUO kidneys. Pathway scores were computed using UCell based on curated gene sets. Red and blue indicate higher and lower relative pathway activity, respectively.

Using the RNA isolated from whole kidney tissues, we performed RNA sequencing (RNA-seq) and unexpectedly found substantial differences in the expression of genes related to multiple oxidation pathways between WT and CD5L KO mice (Fig. 1A). Enrichment scores for various pathways associated with biological oxidations indicated higher oxidative stresses in the kidneys of CD5L KO mice compared to WT mice (Fig. 1B). Similarly, the rCD5L injection also led to expression differences in pathways related to oxidation alongside those related to inflammation and cell injury/cell wounding when tested in CD5L KO mice (Fig. 1C). Enrichment scores suggested that rCD5L injection resulted in activation of antioxidant pathways, while attenuating inflammatory pathways (Fig. 1D).

Histopathological examinations of UUO-kidneys showed that rCD5L-treated mice exhibited significantly milder interstitial fibrosis, a characteristic feature of UUO-induced kidney injury, than PBS-injected controls (Fig. 1E). Furthermore, we found by immunohistochemistry that the levels of representative markers for inflammation (IL-1β, F4/80, and S100A9, a major DAMP) and injury-associated markers (KIM-1, pRIPK3) were lower in the rCD5L-treated mice, while they were higher in the CD5L KO mice compared to the WT mice (Fig. 1F, quantifications are presented in the graphs). Consistent with the RNA-seq results, IHC revealed that CD5L reduced the level of a peroxidized lipid 4-Hydroxynonenal (4-HNE), a common marker of oxidative stress, in UUO kidney (Fig. 1F).

To address the cell types in the kidney where CD5L exerted the effects, we performed single-cell RNA sequencing (scRNA-seq) on kidneys from WT and CD5L KO mice subjected to UUO using pools of four biological replicates per group. A high-quality dataset comprising approximately 153,761 cells was obtained, depicting distinct clusters corresponding to major renal cell types, including immune, epithelial, stromal, and endothelial populations [60] (Supplementary Fig. 2A, B, Supplementary Table 1). The differentially expressed gene (DEG) analysis across all these cell types suggested that endogenous CD5L and/or particularly administered rCD5L modified oxidative stress-related pathways to reduce stress mainly in subtypes of renal proximal tubular epithelial cells (indicated as PT in Figures) (Fig. 1G). In accordance, oxidative stress-related pathways and ferroptosis-associated gene signatures were also attenuated in PT cells. Expectedly, CD5L exerted its effect on inflammation-related pathways in macrophages (Mac) and myofibroblasts (Myofib), while it influenced fibrosis-related pathways in myofibroblasts (Fig. 1G). These trends were preserved at gene levels, when analyzed for different representative genes such as *Sod1*, *Prdx1*, *Prdx6*, *Txnrd1*, *Nqo1*, *Hmox1*, *Srxn1* and *Txn1* (antioxidant), *Gclm* and *Gclc* (GSH synthesis), *Havcr1* (tubular cell injury), *Tgfb1* and *Tgfb3* (fibrosis), *Il1b*, and *Cxcl16* (inflammation), *Fth1*, *Gpx3*, *Cp*, *Slc11a2* and *Slc39a14* (iron metabolism/ferroptosis-associated genes) (Supplementary Fig. 3).

Altogether, in addition to the anti-inflammatory and anti-fibrotic effects expected by the previous observation that CD5L ameliorates AKI through removal of inflammatory organic wastes, these findings suggest that CD5L is associated with reduced oxidative stress, particularly in renal tubular epithelial cells.

### CD5L reduces intracellular ROS-related signals and oxidative stress-associated responses

**Fig. 2:**
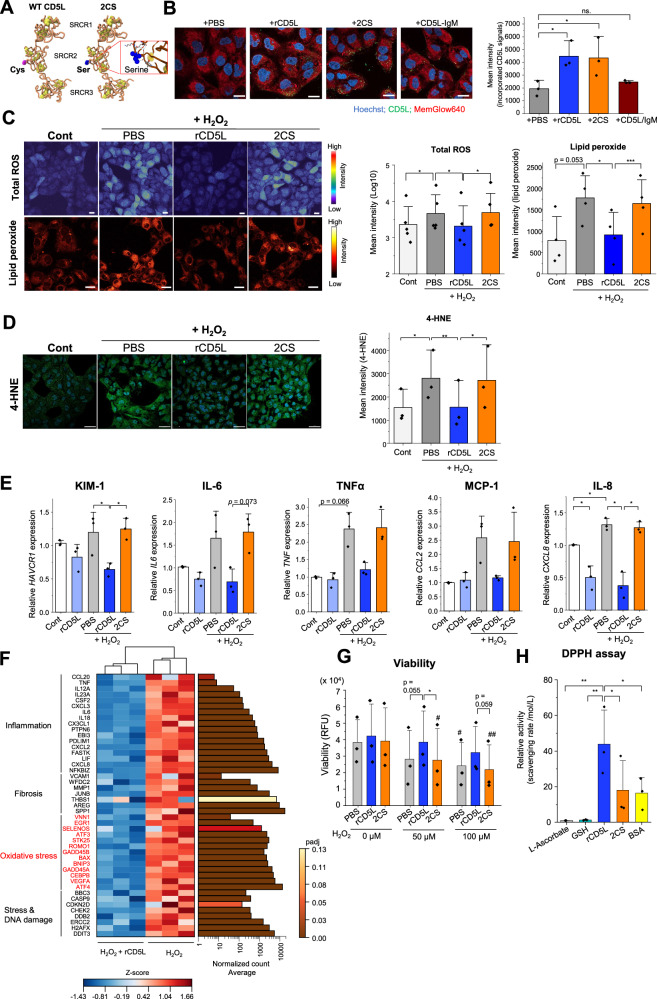
Antioxidant activity of CD5L dependent on the SRCR2 solitary cysteine. **A** Structural models of CD5L and 2CS proteins, created by AlphaFold. Solitary cysteines, cysteines involved in internal disulfide bonds, and the serine substitution in 2CS are indicated. **B** Uptake of rCD5L, 2CS, and the serum-derived CD5L/IgM pentamer complex by HK-2 cells. Cells were stained for CD5L, plasma membrane (MemGlow 640), and nuclei (Hoechst 33342); intracellular CD5L signal intensity is quantified in the graph. Bar, 20 µm. Mean intensity values for intracellular CD5L signals are displayed in the graph. *n* = 3 independent experiments. Statistics: One-way ANOVA with Dunnett’s post hoc test vs. control (+PBS). **C** Intracellular ROS-related fluorescence signals and Liperfluo-detectable lipid peroxide signals in HK-2 cells exposed to H_2_O_2_ (50 µM) with or without rCD5L or 2CS (50 µg/mL each). Cont.; cells without H₂O₂ exposure. Mean signal intensity per well was used as a single data point. *n* = 5 (ROS) or 4 (lipid peroxide) independent experiments. **D** 4HNE staining as a complementary marker of lipid peroxidation-associated oxidative damage in HK-2 cells under the same conditions as in (**C**). Positive signal intensities were quantified as in (**C**). *n* = 3 independent experiments. Bar, 20 µm. Statistics for **C** and **D**: Paired t-tests (paired by experiment) with Holm’s correction were used to compare Cont. vs. PBS, PBS vs. rCD5L, and rCD5L vs. 2CS. **E** mRNA levels of HAVCR1 (KIM-1) and inflammatory genes in HK-2 cells under the indicated conditions. Expression is shown relative to control cells without H_2_O_2_ exposure. *n* = 3 independent experiments. Statistics: Paired t-tests (paired by experiment) with Holm’s correction were used to compare Cont. vs. PBS, Cont. vs. rCD5L, PBS vs. rCD5L, and rCD5L vs. 2CS. **F** Expression changes in inflammation-, fibrosis-, oxidative stress–, and stress-/DNA damage–related genes in HK-2 cells treated with or without rCD5L under H_2_O_2_ stimulation. Heatmap cells show Z-score–normalized expression; adjacent bar graphs indicate mean expression levels, with bar colors representing padj values. **G** HK-2 cell viability after exposure to indicated doses of H_2_O_2_ in the absence (indicated as PBS) or presence of rCD5L or 2CS (50 µg/mL). *n* = 3 independent experiments. Statistics: Two-way ANOVA with Bonferroni’s post hoc test. Statistical significance is indicated by * for comparisons between treatments within the same H_2_O_2_ dose. Bars labeled with # represent significant differences within the same treatment across different H₂O₂ doses. **H** DPPH radical-scavenging assay in a cell-free system. The activity was normalized on a per-molecule basis and expressed relative to L-ascorbate. L-ascorbate and GSH were included as reference compounds, whereas 2CS and BSA were included as protein controls to assess protein-associated background scavenging. *n* = 3 independent experiments. Statistics: One-way ANOVA with Dunnett’s post hoc test vs. rCD5L.

We next asked how CD5L exerts antioxidative functions in kidney. To this end, we induced oxidative stress in HK-2 cells, a proximal tubular epithelial cell line derived from the kidney of a normal human adult male, by treating them with H_2_O_2_ at a concentration within the physiological range found in mammalian serum (50 µM) [61], then exposed the cells to recombinant rCD5L. In addition, we also prepared a mutant form of rCD5L, designated 2CS, in which the solitary cysteine residue in the SRCR2 domain was replaced with serine (Fig. 2A).

Since CD5L is known to enter cells, we used immunocytochemistry to confirm the presence of both rCD5L and 2CS inside HK-2 cells after incubation with the recombinant proteins (Fig. 2B, photos). Quantitative analysis revealed no significant difference in cellular uptake between the two proteins (Fig. 2B, graph). We also observed the presence of internalized rCD5L in the cytosol by immunoblotting after fractionating cytosol from rCD5L-challenged cells (Supplementary Fig. 4). In contrast, the CD5L-IgM pentamer complex was not internalized, likely due to steric hindrance preventing CD5L from binding to scavenger receptors (Fig. 2B).

As shown in Fig. 2C, a challenge of rCD5L reduced intracellular ROS-related signals and Liperfluo-detectable lipid peroxide signals in H_2_O_2_-treated cells, whereas no such reductions were observed with 2CS. Consistent with this result, rCD5L reduced oxidative stress and cell damage, as indicated by decreased staining for 4-HNE following H_2_O_2_ exposure (Fig. 2D). Furthermore, quantitative polymerase chain reaction (qPCR) analysis revealed that mRNA levels of *HAVCR1*, which encodes the injury marker KIM-1, and various inflammatory genes associated with cell damage were significantly lower in rCD5L-treated cells but remained unchanged in 2CS-treated cells under H_2_O_2_ exposure. In addition, for a subset of these genes, rCD5L also lowered basal expression even in the absence of H_2_O_2_ stimulation, indicating that the suppressive effect of CD5L on these markers is not strictly limited to the oxidative stress condition (Fig. 2E). RNA-seq analysis of H_2_O_2_-exposed HK-2 cells further supported broad suppression of expression of the genes related to oxidative stress, inflammation, fibrosis and ferroptosis-associated responses following rCD5L treatment (Fig. 2F). Consistent with these findings, rCD5L—but not 2CS—significantly improved the viability of H_2_O_2_-exposed cells (Fig. 2G).

We assessed whether CD5L exerts direct antioxidant activity using a 2,2-diphenyl-1-picrylhydrazyl (DPPH) assay in a cell-free system, with the results plotted after per-molecule normalization relative to L-ascorbate (Fig. 2H). Under these assay conditions, both 2CS and BSA showed measurable background DPPH-scavenging activity, indicating that the assay includes a nonspecific protein-associated component. However, rCD5L exhibited greater scavenging activity than either 2CS or BSA. Taken together, these findings support the interpretation that the solitary cysteine residue in the SRCR2 domain contributes to the enhanced radical-scavenging activity of CD5L under these assay conditions, although the DPPH signal itself is not exclusively specific to CD5L.

Together, these results support a cysteine-dependent antioxidant activity of CD5L and show that CD5L reduces oxidative stress, inflammatory/stress-associated responses, and cellular injury in this HK-2 cell-based model, with improved cell viability under H_2_O_2_ exposure.

### CD5L augments nuclear Nrf2

**Fig. 3:**
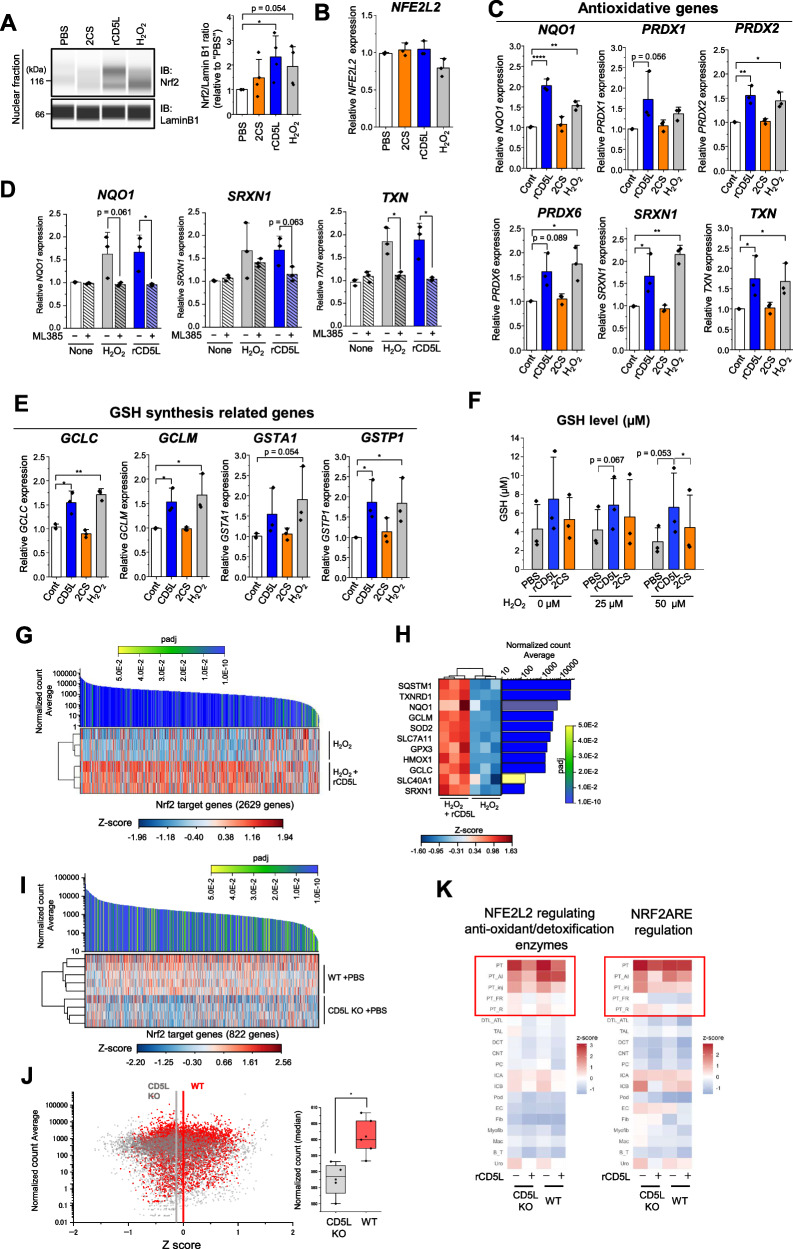
CD5L increases nuclear Nrf2 and enhances antioxidant gene expression. **A** Nuclear Nrf2 detected by immunoblotting in HK-2 cells treated for 2.5 h with PBS (control), 2CS (50 µg/mL), rCD5L (50 µg/mL), or H_2_O_2_ (50 µM). Nrf2 intensity was normalized to lamin B1 and is shown relative to PBS. *n* = 4 independent experiments. Statistics: One-way ANOVA with Dunnett’s post hoc test vs. PBS. Whole blots are shown in Supplementary Fig. 5. **B** mRNA levels of *NFE2L2* (Nrf2). Expression levels relative to those in untreated HK-2 cells (indicated as PBS) are shown. *n* = 3 independent experiments. Statistics: Paired t-tests (paired by experiment) with Holm’s correction were used to compare PBS vs. H_2_O_2_, PBS vs. rCD5L, and rCD5L vs. 2CS. **C** mRNA levels of representative Nrf2-regulated antioxidant genes in HK-2 cells cultured in the presence of rCD5L, 2CS (50 µg/mL each), or H_2_O_2_ (50 µM). Cont.; unstimulated cells. Expression levels are presented relative to those in the control group. n = 3 independent experiments. Statistics: One-way ANOVA with Dunnett’s post hoc test vs. control. **D** Effects of ML385 (2 µM) on H_2_O_2_- and rCD5L-induced expression of representative antioxidant genes in HK-2 cells. n = 3 independent experiments. Statistics: Paired t-tests (paired by experiment) comparing the presence or absence of ML385 within each treatment. **E** mRNA levels of representative Nrf2-regulated GSH synthesis-related genes assessed as in (**C**). **F** Absolute intracellular GSH levels in HK-2 cells exposed to the indicated doses of H_2_O_2_ in the absence (PBS) or presence of rCD5L or 2CS (50 µg/mL each). n = 3 independent experiments. Statistics: Paired t-tests (paired by experiment) with Holm’s correction were performed to compare PBS vs. rCD5L and rCD5L vs. 2CS within the same H₂O₂ dose. **G**, **H** Expression changes in Nrf2 downstream genes based on the Nrf2-ome dataset [84] in HK-2 cells under H_2_O_2_ stimulation with or without rCD5L (*n* = 3 independent experiments). Only differentially expressed genes (DEGs; rCD5L vs. PBS under H_2_O_2_, Benjamini–Hochberg padj < 0.05) were retained, yielding 2629 genes. (**G**) All retained DEGs. (**H**) Representative DEGs. Heatmaps show Z-score–normalized expression; adjacent bar graphs indicate mean expression, with bar colors representing padj values. **I**, **J** Expression of Nrf2 downstream genes based on the Nrf2-ome dataset [84], in kidney tissues from UUO-operated WT and CD5L KO mice. Only DEGs for WT vs. CD5L KO (Benjamini–Hochberg padj < 0.05) were retained, yielding 822 genes. **I** Heatmap showing Z-score–normalized expression, with adjacent bar graphs indicating mean expression and bar colors representing padj values. **J** Distribution of the same genes plotted by normalized expression counts against mean Z-scores. Each dot represents one gene. WT and CD5L KO groups consisted of 5 and 4 biological replicates, respectively. Statistical significance was assessed by the Mann-Whitney U test. **K** Heatmaps showing z-scored median pathway activity of Nrf2-related pathways across major renal cell populations in UUO kidneys. Pathway scores were computed using UCell based on curated gene sets. Red and blue indicate higher and lower relative pathway activity, respectively.

Having observed a marked reduction of cellular oxidative stress by CD5L, we asked whether CD5L might also modulate the activity of Nrf2, which plays a critical role in oxidative stress response by regulating the expression of antioxidant genes involved in ROS scavenging and GSH synthesis. Nrf2 activation is typically achieved by its translocation to the nucleus under oxidative stress [62–64]. Therefore, we assessed nuclear Nrf2 levels by immunoblotting nuclear fraction from HK-2 cells treated with rCD5L, 2CS, or H_2_O_2_ as a positive control. As shown in Fig. 3A (whole blots are presented in Supplementary Fig. 5), rCD5L treatment led to a pronounced increase in nuclear Nrf2 levels, even exceeding that induced by H_2_O_2_ treatment, whereas the total Nrf2 mRNA (*NFE2L2*) levels remained unchanged (Fig. 3B), indicating that CD5L primarily enhances Nrf2 nuclear transport rather than its expression level. In contrast, 2CS showed no augmentation of nuclear Nrf2 accumulation, suggesting that the CD5L-mediated Nrf2 nuclear translocation is also dependent on the reactive cysteine at SRCR2 domain (Fig. 3A).

In accordance with this finding, we detected increased mRNA levels of several antioxidant genes downstream of Nrf2 in response to rCD5L but not to 2CS (Fig. 3C). In line, the rCD5L-induced expression of Nrf2 target genes was downregulated by ML385, a pharmacologic inhibitor commonly used to suppress Nrf2 transcriptional activity (Fig. 3D), consistent with the involvement of Nrf2 in this response. In addition, we found a significant upregulation of mRNA levels for *GCLC, GCLM, GSTA1*, and *GSTP1*, genes involved in GSH synthesis targeted by Nrf2 (Fig. 3E). Consistently, direct quantification showed that the absolute intracellular GSH level was increased by rCD5L, but not by 2CS (Fig. 3F).

RNA-seq analysis on the H_2_O_2_-treated HK-2 cells with or without rCD5L challenge further supported the global upregulation in expression of Nrf2-downstream genes (Fig. 3G). Of those, the expression differences in a representative gene set related to antioxidant-related and GSH synthesis are shown in Fig. 3H. Parallel results were obtained in vivo by RNA-seq analysis of UUO kidneys. Nrf2-downstream genes were broadly upregulated in response to CD5L, as apparent in comparisons between WT and CD5L KO mice, demonstrated by heatmaps (Fig. 3I) or dot blots with a graph showing the mean ± SD (Fig. 3J) of Z-scores. Furthermore, the results of scRNA-seq on the UUO-kidney supported that this event occurred at the tubular epithelial cells (Fig. 3K). Intriguingly, the upregulation of Nrf2 downstream gene expression in UUO kidney by rCD5L administration was not as obvious as the difference between WT and CD5L KO mice (Supplementary Fig. 6).

Together, in addition to showing cysteine-dependent direct radical-scavenging activity in a cell-free assay, CD5L enhances Nrf2-associated antioxidant responses and increases cellular GSH in HK-2 cells, using the reactive cysteine residue in the SRCR2 domain.

### CD5L is associated with reduced cellular ceramide levels, which may contribute to augmented nuclear Nrf2

**Fig. 4:**
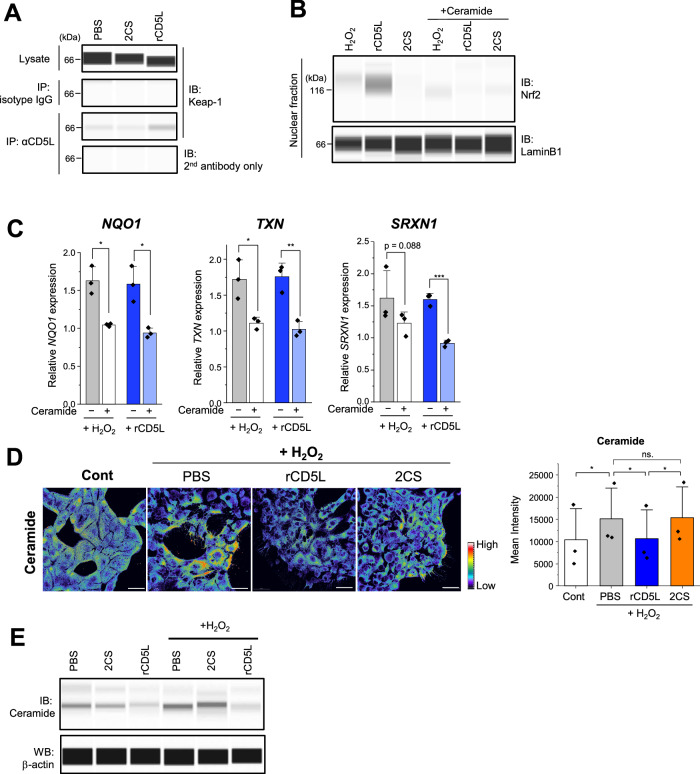
Reduced cellular ceramide is associated with enhanced nuclear Nrf2 in CD5L-treated cells. **A** Co-immunoprecipitation of Keap-1 with rCD5L or 2CS from HK-2 cell lysates using an anti-CD5L antibody or isotype IgG. rCD5L, 2CS, or PBS was added to the lysates before immunoprecipitation. Whole blots are presented in Supplementary Fig. 7A. **B** Effects of ceramide (1 µM) on nuclear Nrf2 in HK-2 cells treated with H_2_O_2_, rCD5L, or 2CS. Nuclear Nrf2 levels were assessed by immunoblotting. Whole blots are presented in Supplementary Fig. 7B. **C** Effects of ceramide (1 µM) on expression of representative Nrf2-downstream genes induced by H_2_O_2_ (50 µM) or rCD5L (50 μg/mL) in HK-2 cells. *n* = 3 independent experiments. **D** Ceramide staining in HK-2 cells exposed to H_2_O_2_ in the absence (PBS) or presence of rCD5L or 2CS (50 μg/mL each). Mean signal intensity per cell was quantified and plotted in the graph. *n* = 3 independent experiments. Statistics: Paired t-tests (paired by experiment) with Holm’s correction were performed to compare Cont. vs. PBS, PBS vs. rCD5L, rCD5L vs. 2CS, and PBS vs. 2CS. **E** Immunoblotting of C16 ceramide-bound proteins, with β-actin as a loading control, in HK-2 cell lysates treated as in (B). Whole blots are presented in Supplementary Fig. 7C.

The well-known pathway of Nrf2 activation under oxidative stress is the oxidation of reactive cysteine residues of Kelch-like-ECH-associated protein 1 (Keap-1). This causes a conformational change of Keap-1, and thereby releases Nrf2, allowing Nrf2 to translocate into the nucleus [62–64]. The deficiency of 2CS in promoting nuclear translocation of Nrf2 implied that the cysteine in the SRCR2 domain of CD5L might form disulfide bond with cysteine residues of Keap-1, mimicking oxidation-induced Nrf2 release from Keap-1. To test this, we performed co-immunoprecipitation of Keap-1 with rCD5L or 2CS in HK-2 cell lysates. As shown in Fig. 4A (whole blots are presented in Supplementary Fig. 7A), however, the amount of Keap-1 co-precipitated with rCD5L was very low and obviously not different from that of negative-control (indicated as PBS). The discrepancy between this result and the high efficiency of rCD5L in promoting Nrf2 nuclear translocation suggests that the CD5L-Keap-1 interaction may have only partial or even no impact on Nrf2 activation. Therefore, we thought that there must be different mechanisms by which CD5L enhances Nrf2 nuclear translocation.

Based on our and others’ previous studies indicating that CD5L can modulate cellular lipid composition [49, 52], we hypothesized that CD5L might reduce cellular ceramide, a bioactive sphingolipid, thereby supporting the nuclear transport of Nrf2 after it is released from Keap-1. This idea is based on the fact that ceramide impairs the nuclear localization of key transport proteins, such as importin-α and Cellular Apoptosis Susceptibility protein, thereby disrupting classical nuclear protein import pathways [65].

To test this possibility, we first analyzed the influence of ceramide on the Nrf2 nuclear translocation. As expected, exogenous administration of ceramide to HK-2 cells abolished the nuclear accumulation of Nrf2 induced by either H_2_O_2_ or rCD5L (Fig. 4B; full blots are presented in Supplementary Fig. 7B). Accordingly, ceramide treatment reduced the expression of various Nrf2 downstream genes that were upregulated by H_2_O_2_ or rCD5L (Fig. 4C).

We then measured cellular ceramide levels in HK-2 cells treated with H_2_O_2_ in the presence of either rCD5L or 2CS, and found that H_2_O_2_ treatment elevated ceramide levels, while rCD5L significantly reduced this increase. In contrast, 2CS failed to lower ceramide levels (Fig. 4D). Immunoblotting for ceramide confirmed that rCD5L reduced C16-ceramide bound proteins in the presence or absence of H_2_O_2_ (Fig. 4E; full blots are presented in Supplementary Fig. 7C). Thus, these results support the possibility that reduced cellular ceramide levels contribute, at least in part, to CD5L-associated augmentation of Nrf2 nuclear translocation.

### CD5L suppresses sphingomyelinase activity and is associated with reduced cellular ceramide

**Fig. 5:**
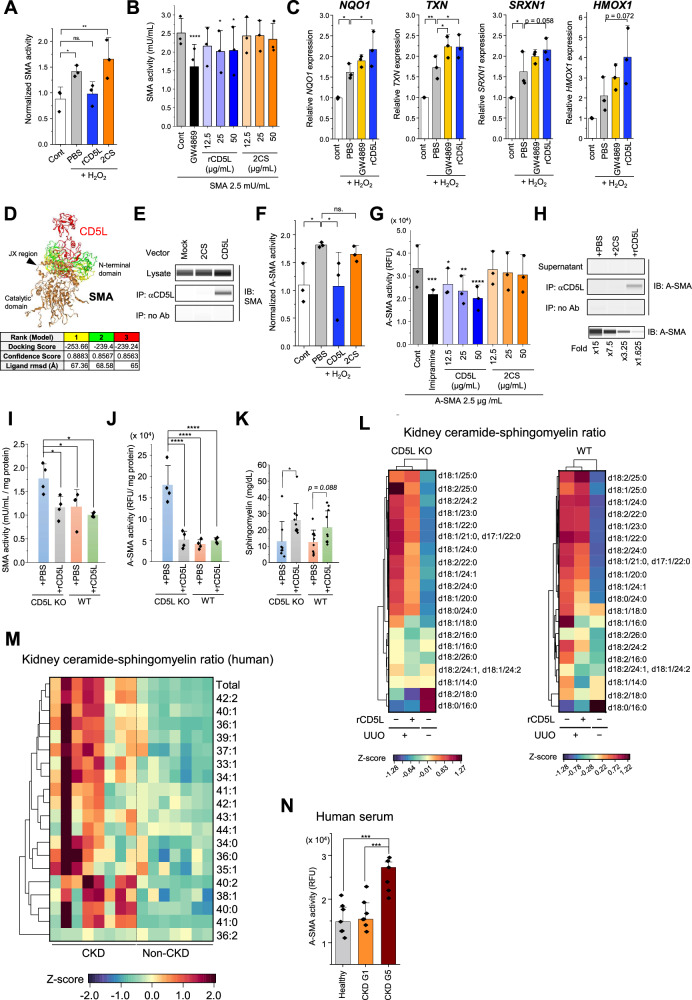
CD5L suppresses sphingomyelinase activity and modulates ceramide-sphingomyelin balance. **A** SMA activity in HK-2 cells exposed to H_2_O_2_ in the absence (PBS) or presence of rCD5L or 2CS (50 μg/mL each). n = 3 independent experiments. Statistics: One-way ANOVA with Dunnett’s post hoc test vs. control (Cont). **B** SMA activity in a cell-free assay system with GW4869 (50 μM) as a positive control. n = 3 independent experiments. Statistics: One-way ANOVA with Dunnett’s post hoc test vs. vehicle control. Bars labeled with * indicate significant differences compared to the control. No significant reduction was observed with 2CS treatment (orange bars). **C** Effects of GW4869 (50 μM) and rCD5L (50 µg/mL) on expression of representative Nrf2 downstream genes in H_2_O_2_ (50 μM)-treated HK-2 cells. n = 3 independent experiments. Statistics: One-way ANOVA with Dunnett’s post hoc test vs. PBS. **D** Docking simulation of CD5L with SMA using the HDOCK server. Structural models of CD5L (AFDB accession: AF-Q9QWK4-F1) and SMA (PDB ID: 5I81) were used as inputs. A total of 100 docking models were generated. The top-ranked docking model is shown, and the docking score, confidence score, and ligand RMSD values for the top three models are summarized in the table. **E** Co-immunoprecipitation of SMA with CD5L or 2CS from HK-2 cell lysates after vector-mediated expression of CD5L or 2CS. Whole blots are presented in Supplementary Fig. 8A. **F** A-SMA activity in culture supernatants from H_2_O_2_ (50 μM)-treated HK-2 cells cultured in the presence or absence of rCD5L or 2CS (50 μg/mL). *n* = 3 independent experiments. Statistics: One-way ANOVA with Dunnett’s post hoc test vs. PBS. **G** Effects of rCD5L and 2CS on A-SMA activity in a cell-free assay, with imipramine (300 μM) as a positive control. n = 3 independent experiments. Statistics: One-way ANOVA with Dunnett’s post hoc test vs. vehicle control. **H** Immunoprecipitation of CD5L or 2CS from culture supernatant of H_2_O_2_ (50 μM)-treated HK-2 cells followed by immunoblotting for A-SMA. Whole blots are presented in Supplementary Fig. 8B. SMA activity in UUO kidneys (**I**) and A-SMA activity in serum (**J**) from UUO-operated WT and CD5L KO mice injected with rCD5L or PBS (*n* = 4–5 per group). Statistics: Two-way ANOVA with Bonferroni’s post hoc test. **K** Serum sphingomyelin levels in UUO-operated WT and CD5L KO mice injected with rCD5L or PBS (*n* = 8–10 per group). Statistics for: Mann–Whitney U test within each genotype. **L** Ratio of ceramide to sphingomyelin species with matched long-chain bases and fatty acid chains in UUO-kidneys from PBS- (*n* = 4) and rCD5L-injected (*n* = 5) mice, with non-UUO kidneys from PBS-injected mice (*n* = 3) shown as controls. Left panels, CD5L KO; right panels, WT. **M** Re-analysis of human kidney lipidomics data [70], showing the ceramide-to-sphingomyelin ratio as in (L) in kidneys from non-CKD (*n* = 7) and CKD (*n* = 8) donors. **N** Serum A-SMA activity in healthy individuals and patients with CKD at G1 and G5 stages (*n* = 7 per group). Statistics: One-way ANOVA with Bonferroni’s post hoc test.

To determine the mechanism of how CD5L regulates the amount of cellular ceramide, we tested possible influence of CD5L on sphingomyelinase (SMA), as ceramide is biosynthesized through hydrolyzing sphingomyelin by SMA. We first examined whether the activity of neutral SMA, the primary cytosolic SMA, is altered by CD5L in HK-2 cells. In line with the fluctuation of ceramide amount (shown in Fig. 4D), H_2_O_2_ treatment increased SMA activity in HK-2 cells, and this increase was effectively blocked by rCD5L (Fig. 5A). A similar decrease in SMA activity was observed in a cell-free assay with rCD5L treatment (Fig. 5B), whereas 2CS failed to significantly reduce SMA activity in either assay (Fig. 5A, B). These findings suggest that CD5L suppresses SMA activity through its SRCR2 cysteine residue, thereby lowering cellular ceramide levels and enhancing Nrf2 nuclear translocation. Consistently, treatment with a SMA inhibitor GW4869 upregulated Nrf2 downstream gene expression, like the effects of rCD5L (Fig. 5C).

Since SMA consists of a catalytic domain and an N-terminal membrane-integral domain—linked by a juxtamembrane region essential for activation [66]—we hypothesized that CD5L might disrupt this interaction. Using the HDOCK protein-protein docking analysis tool [67, 68], we found that CD5L has the potential to bind SMA with high docking scores in a manner that interferes with the association between the catalytic and N-terminal domains (Fig. 5D). To validate this interaction biochemically, we performed co-immunoprecipitation of SMA with CD5L or 2CS in HK-2 cell lysates. SMA co-precipitated with CD5L but not with 2CS (Fig. 5E; whole blots are presented in Supplementary Fig. 8A). Thus, it is likely that CD5L suppressed SMA activity via protein-protein interaction using the reactive cysteine residue at the SRCR2.

Notably, there is a subtype of SMA, acid-SMA (A-SMA), which is secreted from cells. Addition of rCD5L to the culture medium of HK-2 cells also reduced A-SMA activity in the supernatant (Fig. 5F). A similar reduction was observed in a cell-free assay (Fig. 5G). Furthermore, A-SMA was co-precipitated from the HK-2 cell culture supernatant with rCD5L but not by 2CS, supporting the interaction between CD5L and A-SMA, like CD5L and SMA (Fig. 5H and Supplementary Fig. 8B for whole blots).

Similarly, in vivo, SMA activity in kidneys increased upon UUO, and it was significantly higher in CD5L KO mice than WT mice (Fig. 5I, bars indicated “PBS”). Furthermore, rCD5L treatment decreased SMA activity (Fig. 5I, bars indicated “rCD5L”). Parallel results were obtained in secreted A-SMA activity in serum in mice (Fig. 5J). This is consistent with the in vitro observation that the addition of rCD5L to the culture medium of HK-2 cells reduced A-SMA activity in the supernatant (shown in Fig. 5F). Correspondingly, serum sphingomyelin levels increased in mice receiving rCD5L in both WT and CD5L KO mice subjected to UUO (Fig. 5K). Given that ceramide can be biosynthesized from serum sphingomyelins—originating from high- and low-density lipoproteins—and subsequently taken up by renal tubular epithelial cells [69], these findings suggest that CD5L may reduce kidney ceramide levels through suppression of both cellular neutral SMA activity and serum A-SMA activity.

By lipidomic analysis of kidney tissues, we found that the ceramide-to-sphingomyelin ratio was markedly elevated when subjected to UUO in both CD5L KO (left panel) and WT (right panel) mice, indicating a progressive disturbance in ceramide–sphingomyelin balance during UUO progression (Fig. 5L, comparison between 1st lane (UUO+, rCD5L−) and 3rd lane (UUO−) in KO and WT panels). Moreover, rCD5L administration considerably reduced this ratio (Fig. 5L, comparison between 1st lane (UUO+, rCD5L−) and 2^nd^ lane (UUO+, rCD5L+)). Importantly, also in humans, we found that the ceramide-to-sphingomyelin ratio was elevated in the kidney tissue of CKD donors compared to individuals without kidney diseases, by re-analyzing lipidomics data from human kidney samples originally published by Asowata et al. [70] and deposited in Figshare (10.6084/m9.figshare.24991002.v1) (Fig. 5M). Notably, we analyzed sera from patients with advanced CKD (KDIGO stage G5) and age- and sex-matched patients with early CKD (stage G1) or healthy individuals and found that serum A-SMA activity was also increased in humans with advanced-stage CKD (Fig. 5N).

## Discussion

**Fig. 6:**
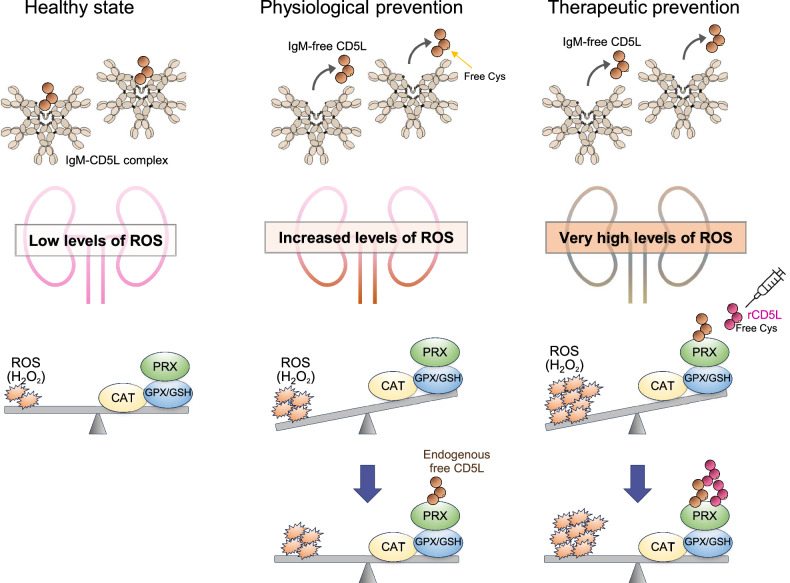
A scheme for CD5L action in healthy and disease states. In a healthy state, the production and elimination of ROS are sufficiently balanced by intracellular redox systems (left). When kidney disease begins, tissue ROS levels increase beyond the capacity of intracellular redox systems to control them. In response, endogenous CD5L dissociates from the IgM pentamer in the serum and enters renal cells, where it cooperates with intracellular systems to help restore redox balance and prevent disease progression (middle). In progressive kidney disease, excess ROS accumulates in tissues, surpassing the regulatory capacity of both intracellular systems and endogenous CD5L. In such cases, rCD5L treatment may help restore redox homeostasis, preventing further disease acceleration (right).

This study identified a kidney-protective mechanism centered on the reduction of cellular oxidative stress by CD5L and further delineated several associated functional observations that may contribute to this effect. Importantly, all the antioxidative functions of CD5L identified in this study depend on the same reactive cysteine residue at SRCR2. The molecular dynamics of CD5L suggest that this exogenous antioxidant pathway is activated upon the progression of disease as a preventive response through the release of CD5L from IgM, when oxidative stress exceeds the capacity of the intracellular redox machinery (Fig. 6, middle). Indeed, we recently reported that the pool of active AIM is released from IgM pentamer depending on the disease states in CKD patients [55]. When oxidative stress becomes excessive and cannot be mitigated by endogenous CD5L, rCD5L treatment provides therapeutic benefits, improves cellular conditions, and protects the kidney (Fig. 6, right).

Through extensive in vitro analyses, we discovered that CD5L exerts a cysteine-dependent intracellular antioxidative effect. The present data support the idea that intracellular CD5L can reduce oxidative stress after cellular uptake, although the precise chemical targets and reaction mechanism remain to be defined.

Since ROS play a role in various physiological processes, intracellular ROS levels and the activity of their regulators must be strictly controlled. This presents challenges in developing drugs targeting ROS- or ROS-mediated signaling pathways. rCD5L may offer an advantage in this regard, as its intracellular concentration appears to be physiologically regulated at multiple checkpoints, including endocytosis, cytosolic translocation from endosomes, and lysosomal degradation. CD5L is endocytosed via scavenger receptors such as CD36, SR-A, and KIM-1, meaning that rCD5L incorporation is limited to cells expressing these receptors. In addition, the rapid excretion of excess CD5L in urine [43, 71] reduces concerns about potential accumulation in the body, an issue often encountered with many drugs. Moreover, unnecessary activation of endogenous CD5L is likely prevented, as CD5L is typically bound to an IgM pentamer in the serum, which inactivates its antioxidant function [53, 54], and the release of active CD5L from the IgM pentamer appears to be disease dependent. Such manifold physiological regulations of CD5L activity likely prevent excessive ROS elimination, thereby avoiding reductive stress and potential tissue damage under physiological conditions.

In addition, we found that CD5L enhances Nrf2-associated antioxidant responses through the SRCR2 cysteine residue, along with its direct antioxidant role. This property of CD5L increases intracellular GSH levels, which is highly beneficial for maintaining cellular homeostasis under oxidative stress. Thus, rCD5L may represent a biologically distinct mode of augmenting Nrf2-associated antioxidant signaling.

It was intriguing that the upregulation of Nrf2 downstream gene expression in UUO kidney by rCD5L administration was not as obvious as the difference between WT and CD5L KO mice when assessed by RNA-seq (Supplementary Fig. 6). According to our current observation that Nrf2 activation is achieved cumulatively by oxidative stress (ROS) and CD5L-ceramide axis, the marked reduction in oxidative stress-associated signals by rCD5L might balance the overall value of Nrf2 activation in the kidney. Certainly, however, further study is required to provide a precise explanation for this observation.

Furthermore, we identified that CD5L downregulates the SMA activity and reduces cellular ceramide levels. Together with the suppressive effects of exogenous ceramide on Nrf2 nuclear accumulation and downstream gene expression, these findings support a functional link between ceramide-related signaling and Nrf2 activation. Accordingly, reduced ceramide levels may contribute, at least in part, to CD5L-associated augmentation of Nrf2 signaling, although the precise temporal sequence remains to be established.

Notably, SMA suppression is also mediated through the SRCR2 cysteine residue. This finding is unexpected but logical, as GSH is a known biological inhibitor of SMA, regulating it in a thiol-dependent manner [72]. Conversely, ceramide metabolism is known to generate a cascade of bioactive lipids that play crucial roles in both normal physiology and disease, including inflammation induction. Although research on the pathological significance of the ceramide-sphingomyelin balance in kidney disease is still ongoing [73, 74], growing evidence suggests that increased SMA activity and dysregulated ceramide–sphingomyelin balance are associated with the progression of cardiovascular diseases and aging, both of which are strongly linked to CKD [75–79]. Moreover, our study is the first to demonstrate increased serum A-SMA activity in UUO mice and humans with CKD. Our findings, indicating reduced serum A-SMA activity by CD5L in parallel with disease improvement in mice, might suggest that rCD5L may offer similar benefits to human patients with CKD.

We acknowledge several limitations of this study. First, the day-12 UUO model used here primarily reflects unilateral obstructive nephropathy with rapid tubulointerstitial injury and fibrosis, rather than the full spectrum of chronic kidney disease. Because the contralateral kidney remained intact and overt renal failure did not develop in this setting, our in vivo findings should be interpreted as evidence for a protective effect of CD5L in UUO-induced fibrotic kidney injury, rather than as direct proof of efficacy across CKD as a whole. Second, the cell-based mechanistic experiments were performed solely in HK-2 cells, which do not fully recapitulate normal renal tubular cell physiology. Therefore, these in vitro findings should be interpreted as mechanistic support obtained in a simplified renal epithelial model and will require validation in additional renal tubular models in future studies. Third, although the lack of SMA inactivation by 2CS suggests the functional association of CD5L with SMA using reactive cysteine residues, additional experiments with SMA mutants (e.g., at the reactive cysteine residues in SMA) are required to precisely identify the binding mode between SMA and CD5L. Fourth, the mechanism for how CD5L, with only a single reactive cysteine residue, can exert a much higher antioxidant efficiency than GSH or sodium L-ascorbate remains to be clarified. A structure-based approach might be of help. Finally, the precise mechanism underlying CD5L dissociation from IgM in such disease states remains unclear. In conclusion, our current findings, together with our previous studies, suggest that CD5L plays a comprehensive role in regulating oxidative stress, chronic inflammation, and renal fibrosis in fibrotic kidney injury by functioning both outside and inside the kidney cells, namely, removing inflammatory and toxic wastes extracellularly while reducing cellular oxidative stress and enhancing antioxidant responses after uptake into target cells. Overall, our findings highlight a new mechanistic avenue for limiting UUO-induced fibrotic kidney injury, with possible relevance to CKD-associated fibrotic and oxidative stress pathways.

## Materials and Methods

### Production and purification of rCD5L

CHO-S cells (Thermo Fisher Scientific, Waltham, MA, USA) stably expressing mouse CD5L were established using Pehx1.1-MCD5L and a mammalian power expression system (Toyobo Co., Ltd., Osaka, Japan). The cells were cultured in CD FortiCHO medium with Anti-Clumping Reagent (Gibco, Grand Island, NY, USA) for 5 days in fed-batch mode using the Middle Scale Bioreactor BCP (ABLE Corporation & Biott Co., Ltd., Tokyo, Japan). Nutrient feeding included CHO CD EfficientFeed Liquid Nutrient Supplement Kit, L-glutamine, and D(+)-glucose (all reagents were obtained from Thermo Fisher Scientific). rCD5L was purified from the culture supernatant using a rat anti-mouse CD5L monoclonal antibody immobilized on HiTrap NHS-activated HP Columns (Cytiva, Marlborough, MA, USA). The bound protein was eluted with 0.1 M Glycine-HCl (pH 2.2) and neutralized with 1 M Tris-HCl (pH 8.5). The protein was then concentrated and buffer-exchanged using Amicon Ultra filter concentrators (Merck Millipore, Burlington, MA, USA) before being stored at −80 °C in PBS. Endotoxin levels were measured using a chromogenic LAL endotoxin detection system (Limulus Color KY Test; Fujifilm Wako Pure Chemical Corporation, Osaka, Japan) following the manufacturer’s protocol. The protein concentration was determined using a Pierce BCA Protein Assay Kit (Thermo Fisher Scientific) according to the manufacturer’s protocol.

### Visualization of 3D structure of CD5L protein and 2CS

The three-dimensional structures of rCD5L and 2CS were modeled using AlphaFold 2, and the data were plotted in the AlphaFold Protein Structure Database. Referenced UniProt ID: Mouse CD5L, Q9QWK4.

### Mice

This study was conducted in strict accordance with the Guide for the Care and Use of Laboratory Animals from the National Institutes of Health. All mouse strains used for the analysis had a C57BL/6J background (CLEA Japan, Tokyo, Japan) and were maintained under specific pathogen-free conditions with recommended husbandry practices. The humane endpoints were defined as decreased spontaneous movement, abnormal respiration or pulse, reduced body temperature, altered posture such as lying down on the belly, and decreased response to external stimuli. The animals meeting these criteria were euthanized by releasing blood through the abdominal aorta under isoflurane anesthesia. All experimental procedures were performed on 8 to 9-week-old male mice.

### UUO-induced renal fibrosis model

The procedure was performed as previously described [59]. Briefly, animals were anesthetized with isoflurane, and butorphanol tartrate (1 mg/kg, 5 mL/kg) was administered subcutaneously for analgesia. The surgical area was disinfected with 70% alcohol, and the left kidney’s ureter was exposed and ligated at two points using a thread. The central portion of the ureter was then excised. Mice received daily injections of rCD5L (0.5 mg/mouse/injection) or control PBS. Twelve days post-surgery, the kidneys were harvested following heparin (10 U/mL) perfusion.

### Histological analysis and immunohistochemistry

Kidney tissues were fixed in 4% paraformaldehyde (PFA), embedded in paraffin, and sliced into 4-μm sections. These sections were stained with AZAN for histopathological analysis. For immunohistochemistry, the sections were first deparaffinized and subjected to antigen retrieval using an L.A.B. solution. Endogenous peroxidase activity was blocked with 3% hydrogen peroxide, followed by treatment with a G-Block solution (Genostaff, Tokyo, Japan). The sections were then incubated with primary antibodies, including a rabbit anti-mouse CD5L polyclonal antibody (developed in-house) and antibodies against 4-HNE (Bioss, Woburn, MA, USA; bs-6313R), cleaved-IL-1β (Asp116; active form, Invitrogen, Waltham, MA, USA; PA5105048), F4/80 (clone: A3-1; Abcam, Cambridge, UK; ab6640), S100A9 (R&D Systems, Minneapolis, MN, USA; AF2065), Kim-1 (R&D Systems, AF1817), and RIP3 (pRIPK3) (clone: 2D7; Abcam, ab205421). A HITOFINE Simple Stain Mouse MAX-PO (R, M, G, or Rat) (Nichirei Biosciences, Tokyo, Japan) was used as the secondary antibody. After staining, diaminobenzidine tetrahydrochloride (DAB) was applied for visualization, and the sections were counterstained with HE. For multiplex immunofluorescence of UUO kidney sections, similarly prepared sections were incubated with antibodies against CD5L, F4/80, and LRP2 (Abcam, ab76969), followed by species-appropriate Alexa Fluor 488-, 546-, and 647-conjugated secondary antibodies (Molecular Probes, Waltham, MA, USA). Nuclei were counterstained with Hoechst 33342 (Invitrogen), and autofluorescence was quenched using the Vector TrueVIEW Autofluorescence Quenching Kit (Vector Laboratories, Newark, CA, USA). The standard tissue sections were imaged using a VS200 slide scanner (Olympus, Tokyo, Japan) equipped with a 20x objective lens. Digital image analysis was performed using HALO software (Indica Labs, Albuquerque, NM, USA).

### RNA-sequencing

Total RNA was extracted from kidney tissue or HK-2 cells, ensuring an RNA integrity number (RIN) ≥ 7 before proceeding with library preparation. RNA-seq libraries were constructed using random hexamer primers and sequenced by Novogene Co., Ltd. (Beijing, China) using the Illumina NovaSeq PE150 platform, generating 150-base-pair paired-end reads. To ensure high-quality sequencing data, adapters and low-quality bases were removed using Trimmomatic. The clean reads were then aligned to the appropriate reference genome using HISAT2 (mouse: GRCm38/mm10; human: GRCh38). Gene expression levels were quantified using featureCounts, while differential expression analysis was conducted using DESeq2. Functional annotation and pathway enrichment analysis were performed using ClusterProfiler and Gene Set Enrichment Analysis (GSEA, version 4.3.2).

### Single-cell RNA-sequencing

Single-cell RNA sequencing (scRNA-seq) was performed on kidneys from WT and CD5L KO mice subjected to UUO. Four biological replicates per group were pooled and processed using the 10x Genomics Chromium Flex platform (10x Genomics, Pleasanton, CA, USA) in a single batch. Libraries were prepared with the Chromium Mouse Transcriptome Probe Set for Flex Gene Expression chemistry and sequenced on an Illumina platform (Illumina, San Diego, CA, USA). Raw sequencing data were processed using Cell Ranger (v9.0.1) with the reference transcriptome refdata-gex-mm10-2020-A. The resulting feature–barcode matrices were imported into R (v4.5.1) and analyzed using Seurat (v4.3.0) in RStudio. Cells with fewer than 400 or more than 7,500 detected genes or >10% mitochondrial RNA content were excluded. Gene expression data were normalized and log-transformed, and highly variable genes were identified using FindVariableFeatures. Data were scaled and subjected to principal component analysis (PCA) using the top 30 principal components. Potential doublets were identified and removed using DoubletFinder (v2.0.6) with an expected doublet rate of 10%, and optimal pK values determined by find.pK. Following doublet removal, stringent quality control was applied to retain only cells with 500 < nFeature_RNA < 6000, 500 < nCount_RNA < 15,000, and <5% mitochondrial content. After filtering, a total of 153,761 high-quality cells were retained for downstream analysis. The four datasets were merged and normalized, and the top 5000 highly variable genes were selected. Data were scaled while regressing out total UMI counts and mitochondrial percentage. Dimensionality reduction was performed by PCA (50 components), followed by construction of a shared nearest neighbor (SNN) graph using cosine distance (k = 100). Cells were clustered using the Louvain algorithm at a resolution of 1.2, and Uniform Manifold Approximation and Projection (UMAP) was used for visualization (min.dist = 0.05, spread = 0.8, n.epochs = 2000). Cell type annotation was performed using AddModuleScore based on curated marker gene sets (Supplementary Table 1), and clusters were assigned to the cell type with the highest module score. Visualization of annotated clusters was conducted using the plot1cell framework [80].

### HK-2 cell culture

HK-2 cells (purchased from ATCC, Manassas, VA, USA; CRL-2190; RRID: CVCL_0302) were cultured in DMEM (Gibco), supplemented with 10% (v/v) fetal bovine serum (FBS; Thermo Fisher Scientific) and 20 µg/mL Gentamicin (Thermo Fisher Scientific), at 37 °C in a humidified atmosphere containing 5% CO_2_. One day before using the cells for experiments, the medium was replaced with glutamine-depleted medium (DMEM, High Glucose; Fujifilm Wako Pure Chemical, Osaka, Japan), supplemented with 10% (v/v) FBS and 20 µg/mL Gentamicin, and further incubated overnight. A reference STR profile is available via the ATCC STR database; no additional STR profiling was performed in our laboratory for this study. Mycoplasma testing results for the purchased lot were obtained from the supplier’s Certificate of Analysis.

### Immunocytochemistry

HK-2 cells were cultured in multi-well glass-bottom dishes (Matsunami Glass, Osaka, Japan) for immunocytochemical analysis. The cells were first fixed with 4% PFA and permeabilized using 0.1% Triton-X-100. To minimize non-specific staining, a G-Block solution was applied before incubation with primary antibodies. The primary antibodies included a rabbit polyclonal anti-mouse CD5L (developed in-house), 4-HNE, and ceramide (clone: MID 15B4, Sigma-Aldrich, St. Louis, MO, USA). Appropriate species-specific secondary antibodies were used for detection in each experiment. Nuclear staining was performed using Hoechst 33342. The stained cells were visualized under an LSM980 confocal microscope (Carl Zeiss, Oberkochen, Germany), and fluorescence intensity was quantified using ZEN software. A region of interest (ROI) was defined for ≥ 20 cells per image to ensure consistent analysis.

### Cell viability assay

The viability of HK-2 cells following experimental treatments was assessed using the PrestoBlue (Invitrogen). After treatment, the culture supernatant was replaced with a medium containing 10% PrestoBlue reagent, and the cells were incubated at 37 °C in a 5% CO₂ atmosphere for 1 h. Fluorescence intensity was then measured at an excitation wavelength of 560 nm and an emission wavelength of 590 nm to determine cell viability.

### Total ROS and lipid peroxidation detection

After the HK-2 cells were treated with 50 µM H_2_O_2_ for 2 h, the medium was replaced with fresh culture medium containing either mouse rCD5L, 2CS (50 µg/mL), or an equivalent volume of PBS. The cells were then incubated for an additional 3 h. Cells were then stained with the ROS Assay Kit - Photo-oxidation Resistant DCFH-DA (Dojindo, Kumamoto, Japan) at a concentration of 10 µM for 30 min. After washing with HBSS (+), the cells were imaged using an LSM980 confocal microscope under low laser power conditions (excitation: 493 nm; emission: 513 nm, laser power: 0.05%, averaging: 2, gain: 900 V, LSM plus). Lipid peroxidation was assessed by staining cells with Liperfluo (Dojindo) at a concentration of 20 µM for 15 min. The stained cells were then observed using the same confocal system under Lambda mode (excitation: 488 nm; averaging: 4). The mean fluorescence intensity was quantified using ZEN software by defining an ROI of at least 20 cells per image in the acquired confocal images.

### Quantitative RT-PCR

Total RNA was extracted from the samples using the ReliaPrep RNA Miniprep System (Promega, Madison, WI, USA). Complementary DNA (cDNA) synthesis was performed using the SuperScript IV VILO Master Mix (Thermo Fisher Scientific). Quantitative evaluation of mRNA expression was conducted using the QuantStudio 3 Real-Time PCR system (Thermo Fisher Scientific) with Power SYBR Green PCR Master Mix (Thermo Fisher Scientific) and the ΔΔCT method. The oligonucleotide sequences used for the analysis are provided in Supplementary Table 2.

### Intracellular GSH measurement

For the intracellular GSH assay, HK-2 cells were maintained in D-MEM (high glucose, phenol red; Wako, 045-30285). HK-2 cells were lysed using cold Mammalian Cell Lysis Buffer (Abcam). Proteins were removed by deproteinization and neutralization using a TCA Deproteinization Sample Preparation Kit (AAT Bioquest, Pleasanton, CA, USA). Intracellular GSH levels in the lysate supernatants were directly quantified using the GSH/GSSG Ratio Detection Assay Kit II (Fluorometric) (Abcam) according to the manufacturer’s instructions. Fluorescence intensity (excitation: 490 nm; emission: 520 nm) was measured, and absolute GSH amounts were calculated based on a standard curve.

### DPPH antioxidant assay

The direct antioxidant activity of the tested compounds was evaluated using the DPPH Antioxidant Assay Kit (Dojindo) according to the manufacturer’s protocol. Absorbance was measured at 517 nm using a microplate reader. The radical scavenging activity (%) of each sample was calculated as an indicator of antioxidant activity. The values were normalized to the molar concentration corresponding to 250 μg/mL for each molecule and plotted as relative values compared to sodium L-ascorbate.

### Cell Fractionation

HK-2 cells were harvested and fractionated into cytoplasmic, membrane/organelle, and nuclear fractions using the SF PTS Kit (GL Science, Tokyo, Japan) according to the manufacturer’s protocol. Following fractionation, each fraction was concentrated and buffer-exchanged to PBS using Amicon Ultra-0.5 mL centrifugal filters (10 kDa cutoff, Merck Millipore). To detect nuclear Nrf2, the cell pellet was fractionated into cytoplasmic, nuclear, and insoluble nuclear fractions using the LysoPure™ Nuclear and Cytoplasmic Extractor Kit (Fujifilm Wako Pure Chemical) according to the manufacturer’s protocol, with the addition of a protease inhibitor cocktail. The processed fractions were then used for immunoblotting.

### Immunoblotting (WES analysis)

Immunoblotting was performed using the ProteinSimple Wes system (ProteinSimple, San Jose, CA, USA) according to the user guide. Primary antibodies and dilutions were as follows: anti-Nrf2 (Proteintech, Rosemont, IL, USA;16396-1-AP; 1:50), anti-Keap-1 (Proteintech, 10503-2-AP; 1:50), anti-A-SMA (Bioss, BS-6318R; 1:100), anti-SMA (Abcam, ab172793; 1:100), anti-Ceramide antibody (Sigma-Aldrich, C8104-50TST; 1:40), anti-GAPDH (Proteintech, 10494-1-AP; 1:100), anti-Lamin B1 (Abcam, ab16048; 1:100), anti-β-Actin (Cell Signaling Technology, Danvers, MA, USA; 4970S; 1:200). Secondary antibodies were selected based on the host species of the primary antibodies and applied according to the ProteinSimple Wes system protocol.

### Co-immunoprecipitation

Co-immunoprecipitation (Co-IP) was conducted using the Universal Magnetic Co-IP Kit (Cat. no. 54002; Active Motif, Carlsbad, CA, USA). To evaluate the interaction between CD5L and SMA, HK-2 cells were transfected with CD5L and 2CS expression vectors (in pCAGGS) or a mock vector using Lipofectamine 2000 (Invitrogen) and incubated for 48 h before harvesting. The cells were lysed using the Complete Whole-cell Lysis Buffer provided in the kit. The lysates were clarified by centrifugation, and the supernatant was incubated with or without an anti-mouse CD5L antibody or a control rabbit IgG antibody at 4 °C for 4 h. The samples were then mixed with magnetic Protein G beads (included in the kit). Following incubation, the beads were boiled in 1x Sample Buffer for analysis using the ProteinSimple WES system, and the co-precipitation of SMA was assessed. To evaluate the association between Keap-1 and CD5L, rCD5L or 2CS was added to HK-2 cell lysates along with phosphatase and deacetylase inhibitors. The mixtures were incubated with gentle rotation at 4 °C overnight. Subsequently, rCD5L or 2CS was immunoprecipitated using an anti-CD5L antibody, and the precipitates were analyzed for Keap-1 using the WES system. In addition, the interaction between A-SMA and CD5L was investigated using the HK-2 culture supernatant, where rCD5L or 2CS was added before immunoprecipitation with an anti-CD5L antibody. The same procedure used for Keap-1 and CD5L was followed. The precipitates were analyzed for A-SMA content using the WES system. Since A-SMA was detectable in the culture supernatant, the supernatant was concentrated for A-SMA detection through immunoblotting.

### Lipidomics analysis

Lipidomics were performed following established protocols with slight modifications [81, 82]. Briefly, 200 μL of suspension from freeze-crushed mouse kidney tissue in methanol containing internal standards was mixed with 100 μL of chloroform and incubated at 20 °C for 20 min. An additional 20 μL of water was then added, and the sample was incubated for another 20 min at 20 °C before undergoing centrifugation at 1670 x *g* for 10 min. The supernatants from mouse kidney samples were collected and transferred to LC vials. Lipidomic analysis was conducted using a quadrupole time-of-flight (Q-TOF) mass spectrometer (TripleTOF 6600; Sciex, Framingham, MA) coupled with an ACQUITY UPLC system (Waters, Milford, MA), following previously described methodologies [83]. Publicly available human kidney lipidomics data reported by Asowata et al. [70] and deposited in Figshare were reanalyzed. In the original dataset, the control group was analyzed here as the non-CKD group, whereas the impaired renal function group was analyzed as the CKD group.

### Measurement of sphingomyelinase activity

HK-2 cells were sonicated using a BIORUPTER II (Power: High, 30 s x 20 cycles, 30-sec interval, BM Equipment, Tokyo, Japan), followed by centrifugation at 4 °C. The resulting supernatant was collected and used as the sample. Neutral SMA activity was determined using the Neutral Sphingomyelinase Activity Assay Kit (Echelon Biosciences, Salt Lake City, UT, USA). The reaction mixture consisted of the SMA standard solution, the sample, and Choline Chloride (positive control), and absorbance was measured at 595 nm. For the cell-free assay, an equal volume of the test solution was mixed with 5 mU/mL of the SMA standard solution and measured as a sample. The activity of A-SMA in culture supernatants or sera (applied without dilution) was assessed using the Amplite® Fluorimetric Acidic Sphingomyelinase Assay Kit (AAT Bioquest) in a procedure similar to the neutral SMA activity assay. To prepare samples for the cell-free assay, an equal volume of the test solution was added to a dilution of 0.5 μg/mL of recombinant human acidic sphingomyelinase (R&D Systems) in MES Buffer, and the mixture was used as a test sample. Serum sphingomyelin levels were measured using a Sphingomyelin Colorimetric Assay Kit (Cayman Chemical, Ann Arbor, MI, USA) according to the manufacturer’s instructions. Briefly, 10 µL of mouse serum was applied to each well, followed by the addition of 100 µL reaction mixture, incubation for 60 min at room temperature, and absorbance measurement at 590 nm.

### Statistics and reproducibility

All statistical analyses were performed using OriginPro 2024b (OriginLab, Northampton, MA, USA) and Bell Curve for Excel (Social Survey Research Information Co., Ltd., Tokyo, Japan). No statistical methods were used to predetermine sample size. For animal experiments, group sizes were chosen based on prior experience with the UUO model and feasibility, and are comparable to those commonly used in the field. For in vitro experiments, sample sizes were based on independently repeated experiments (biological replicates) as stated in the figure legends. In the UUO study, mice were allocated to PBS or rCD5L within each genotype in a balanced manner; no formal randomization was performed, and surgeries/injections and downstream sample processing were performed in an alternating order across groups to minimize batch effects. Data distribution was assessed using the Shapiro–Wilk test, and homogeneity of variances was assessed using Levene’s test (or the Brown–Forsythe test, as appropriate). For two-group comparisons, Welch’s two-sided t-test was used when data were approximately normally distributed (without assuming equal variances). When normality assumptions were not met, the Mann–Whitney *U* test was used for two-group non-parametric comparisons. When multiple pairwise comparisons were performed, P values were adjusted using the Holm method, as specified in the figure legends. For comparisons involving more than two groups, one-way ANOVA was used, followed by Bonferroni’s *post hoc* test; when comparisons were made against a single control group, Dunnett’s *post hoc* test was used. Two-way ANOVA with Bonferroni’s *post hoc* test was used to analyze the effects of two independent variables. The exact tests used for each figure are specified in the figure legends. Statistical significance was determined based on the following thresholds: *P < 0.05, **P < 0.01, ***P < 0.001, #*P* < 0.05, and ##*P* < 0.01. Violin plots displayed individual data points, while bar graphs represented the mean along with positive standard deviation and individual data points. Box plots depicted the median (center line) and interquartile range (25–75%); whiskers extend to the most extreme data points within 1.5×IQR (Tukey), with individual data points overlaid. For UUO experiments, animals that reached the predefined humane endpoints before the planned endpoint were excluded from the analysis. For in vitro oxidative-stress assays, experiments were excluded if the H_2_O_2_-only positive control did not show an increase in oxidative-stress readouts compared with untreated controls, or if extensive cell detachment prevented imaging/quantification. These criteria were predefined before data acquisition and applied uniformly across groups. Blinding was not performed. Image quantification was performed using predefined analysis settings in HALO by an investigator who was not involved in the experiments.

## Supplementary information

Supplementary Information

Supplementary Table 1

Supplementary Table 2

## Data Availability

The lipidomics dataset used in this study has been deposited in figshare under: 10.6084/m9.figshare.31524346. The bulk RNA-seq datasets generated from UUO kidney tissues and HK-2 cells, as well as the single-cell RNA-seq dataset generated in this study, have been deposited in the Gene Expression Omnibus (GEO) under accession numbers GSE324326, GSE324328, and GSE324542, respectively. All other data generated or analyzed during this study are included in this article and its Supplementary Materials file, or are available from the corresponding author upon reasonable request. No custom code was developed for this study.
