# Supplementary material for: A kidney-protective mechanism via cellular oxidative stress reduction induced by CD5L protein
Source: Cell Death Discov. 2026 May 23;12:311. doi: 10.1038/s41420-026-03171-2 (PMC13377098; doi:10.1038/s41420-026-03171-2)
Supplement: Supplementary file 1 — Supplementary Information [file 41420_2026_3171_MOESM1_ESM.pdf]

Supplementary Information
for

A kidney-protective mechanism via cellular oxidative stress reduction induced by CD5L protein

Kai Kudo, Takashi Ikeda, Kazutaka Ikeda, Natsumi Maehara, Aika Hirota, Yuri Yoshikawa, Haruka Mori, Masumi Takayama, Keisuke Yasuda, Tetsushi Tezuka, Toshio Takagi, Satoko Arai, Toru Miyazaki

This file includes

Supplemental Fig. 1 to 8

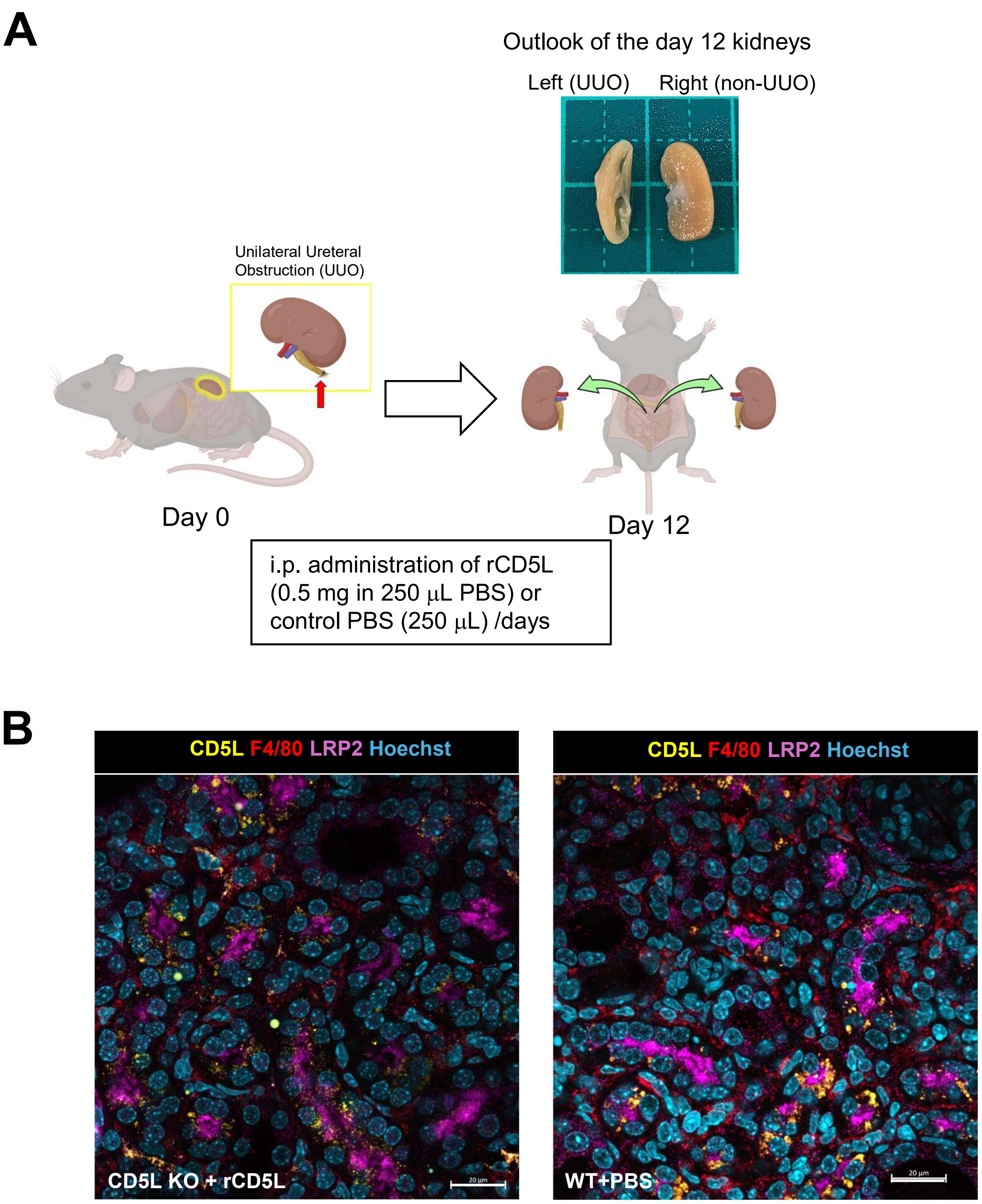

Supplemental Fig.1 Administration of rCD5L to UUO mouse models.
(A) Experimental layout for UUO. The left ureter of mice (male, 9 weeks of age) was ligated, followed by intraperitoneal injection of rCD5L (500 µg in 250 µL of PBS) or PBS (250 µL) daily. On day 12, kidneys were perfused with PBS and harvested for further analyses. Groups: CD5L KO + PBS, CD5L KO + rCD5L, WT + PBS, and WT + rCD5L. **(B)** Multiplex immunohistochemistry of UUO kidneys. Representative cortical images from UUO-operated CD5L KO + rCD5L kidney (left) and WT + PBS kidneys (right). CD5L (yellow), F4/80 (red), LRP2 (magenta), Hoechst (cyan). Scale bar, 20 µm.

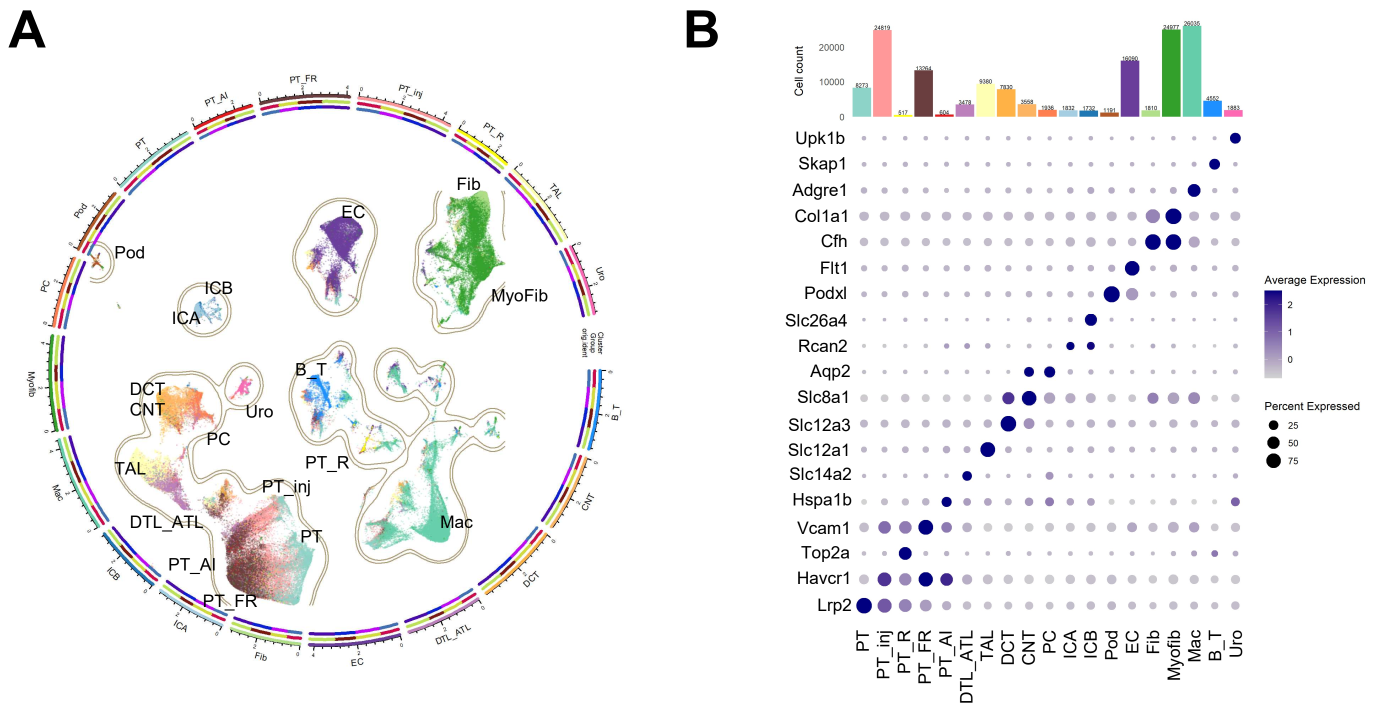

Supplemental Fig. 2: scRNA-seq analysis of UUO kidney. (A) Single-cell atlas of UUO kidneys. UMAP plot (center) showing 153,761 cells profiled from kidneys of 16 mice (CD5L KO + PBS, CD5L KO + rCD5L, WT + PBS, WT + rCD5L). Circular layout (periphery) indicates the $\log_{10}$-transformed cell counts for each population (outer ring: 19 major cell types; inner ring: proportion of each group within each cell type). PT: proximal tubule; PT_inj: injured PT; PT_R: repairing PT; PT_FR: failed-repair PT; PT_AI: acute-injury PT; DTL: descending thin limb of Henle's loop (LoH); ATL: ascending thin limb of LoH; TAL: thick ascending limb of LoH; DCT: distal convoluted tubule; CNT: connecting tubule; PC: principal cell of collecting duct; ICA: type A intercalated cell; ICB: type B intercalated cell; Pod: podocyte; EC: endothelial cell; Fib: fibroblast; Myofib: myofibroblast; Mac: macrophage; B_T: lymphocyte; Uro: urothelial cell. **(B)** Cell-type marker expression and cell count distribution. Dot plot showing the expression patterns of cell type–specific marker genes across annotated cell clusters, alongside a bar plot showing the number of cells per annotated type. In the dot plot, circle diameter corresponds to the proportion of cells expressing the indicated gene, and color intensity reflects the average expression level across all cells of that type.

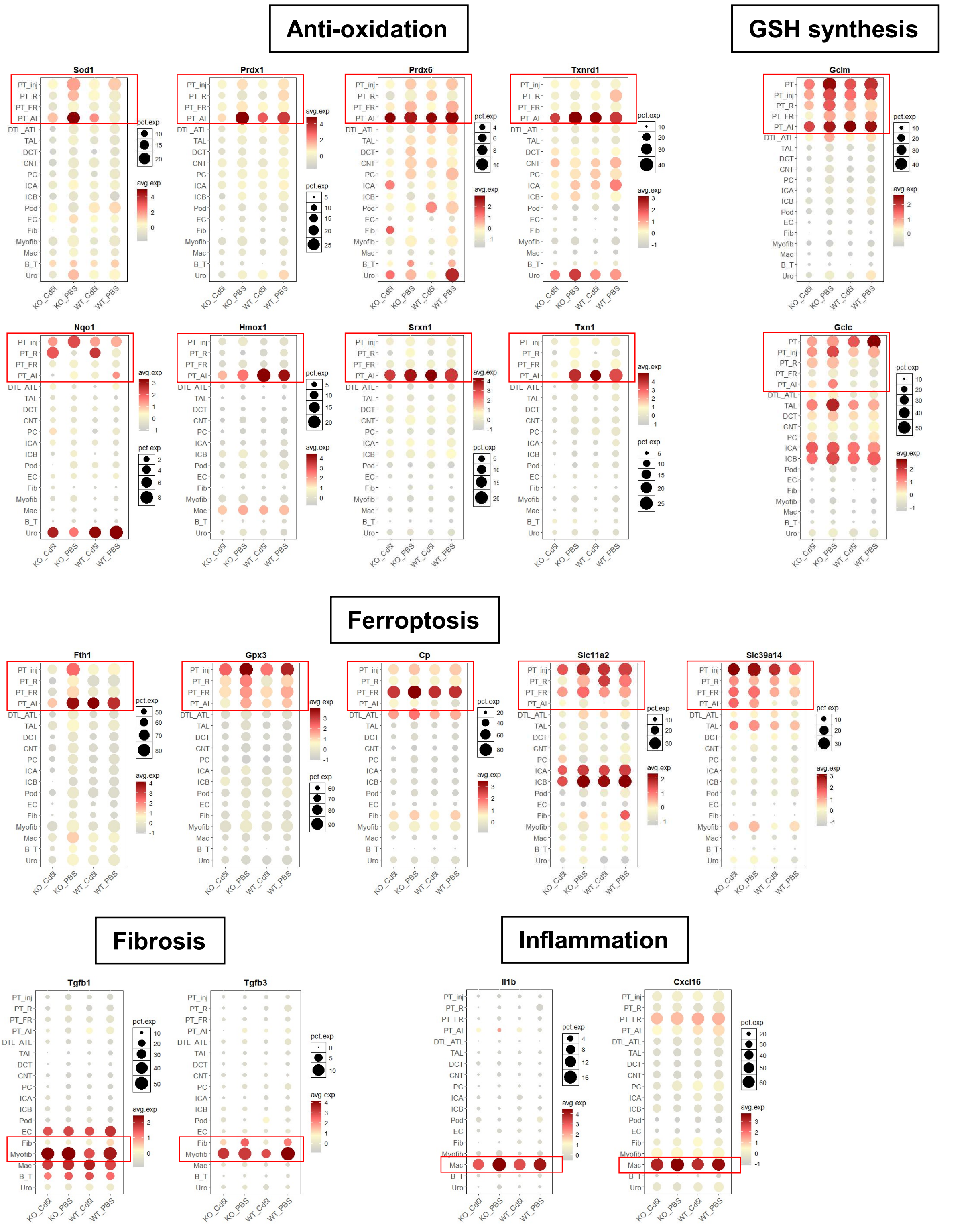

Supplemental Fig. 3. Change in gene expression by CD5L in different cell types in UUO kidney. Bubble plot of representative genes related to Anti-oxidation, GSH synthesis, ferroptosis, fibrosis, and inflammation across cell types. Bubble size represents the proportion of cells expressing the gene; color indicates average expression level. Abbreviations: (Y-axis) PT: proximal tubule; PT_inj: injured PT; PT_R: repairing PT; PT_FR: failed-repair PT; PT_AI: acute-injury PT; DTL: descending thin limb of Henle's loop (LoH); ATL: ascending thin limb of LoH; TAL: thick ascending limb of LoH; DCT: distal convoluted tubule; CNT: connecting tubule; PC: principal cell of collecting duct; ICA: type A intercalated cell; ICB: type B intercalated cell; Pod: podocyte; EC: endothelial cell; Fib: fibroblast; Myofib: myofibroblast; Mac: macrophage; B_T: lymphocyte; Uro: urothelial cell. (X-axis) KO: CD5L KO, _Cd5l: treated with rCD5L, _PBS: treated with PBS.

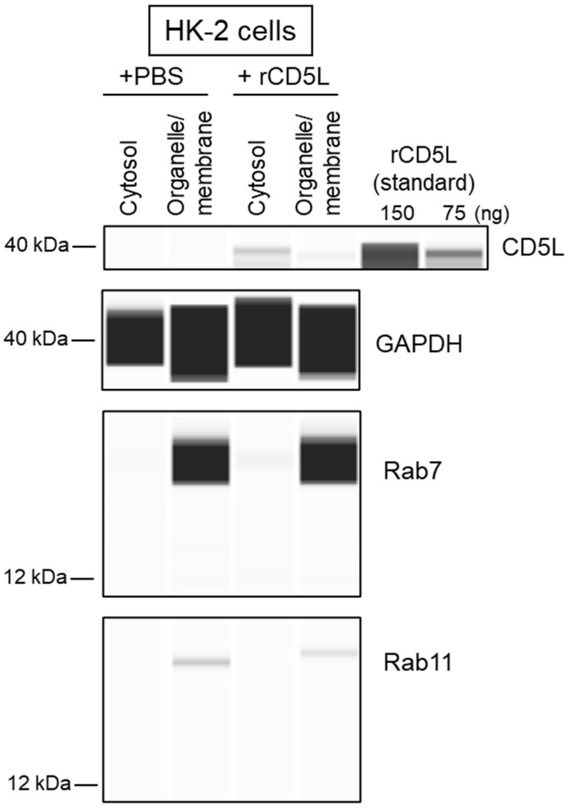

Supplemental Fig. 4. Localization of incorporated rCD5L at cytosol. Proteins of cytosol and organelle/membrane fractions isolated from HK-2 cells challenged with rCD5L (50 µg/mL) or PBS for 1.5 h in culture was assessed for CD5L, GAPDH (control for all fractions), Rab7 and Rab11 (controls for organelle/membrane fraction) by immunoblotting. The amount of protein loaded per lane was 0.16 µg (cytosol) and 0.25 µg (organelle/membrane). rCD5L protein (150 or 70 ng/lane) was loaded as a standard.

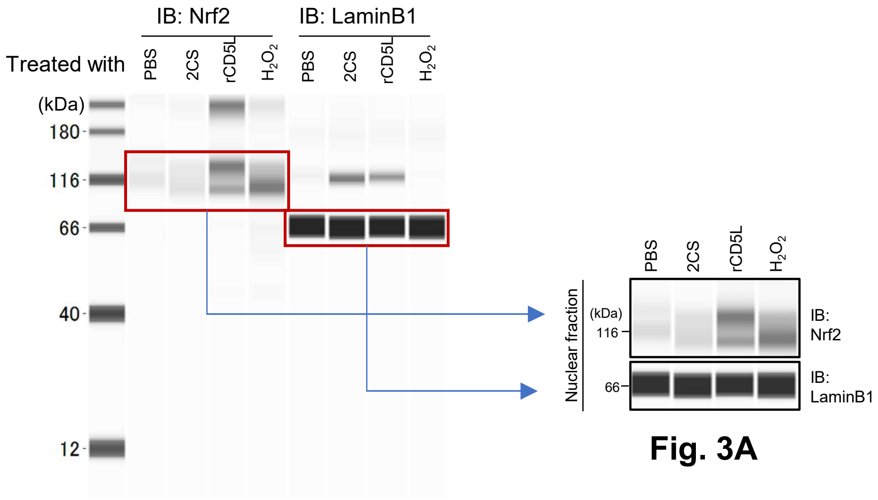

Supplemental Fig. 5: The whole blot of nuclear NRF2 immunoblotting (related to Fig. 3A). Immunoblotting of Nuclear Nrf2 or laminin B1 from HK-2 cells treated with indicated item.

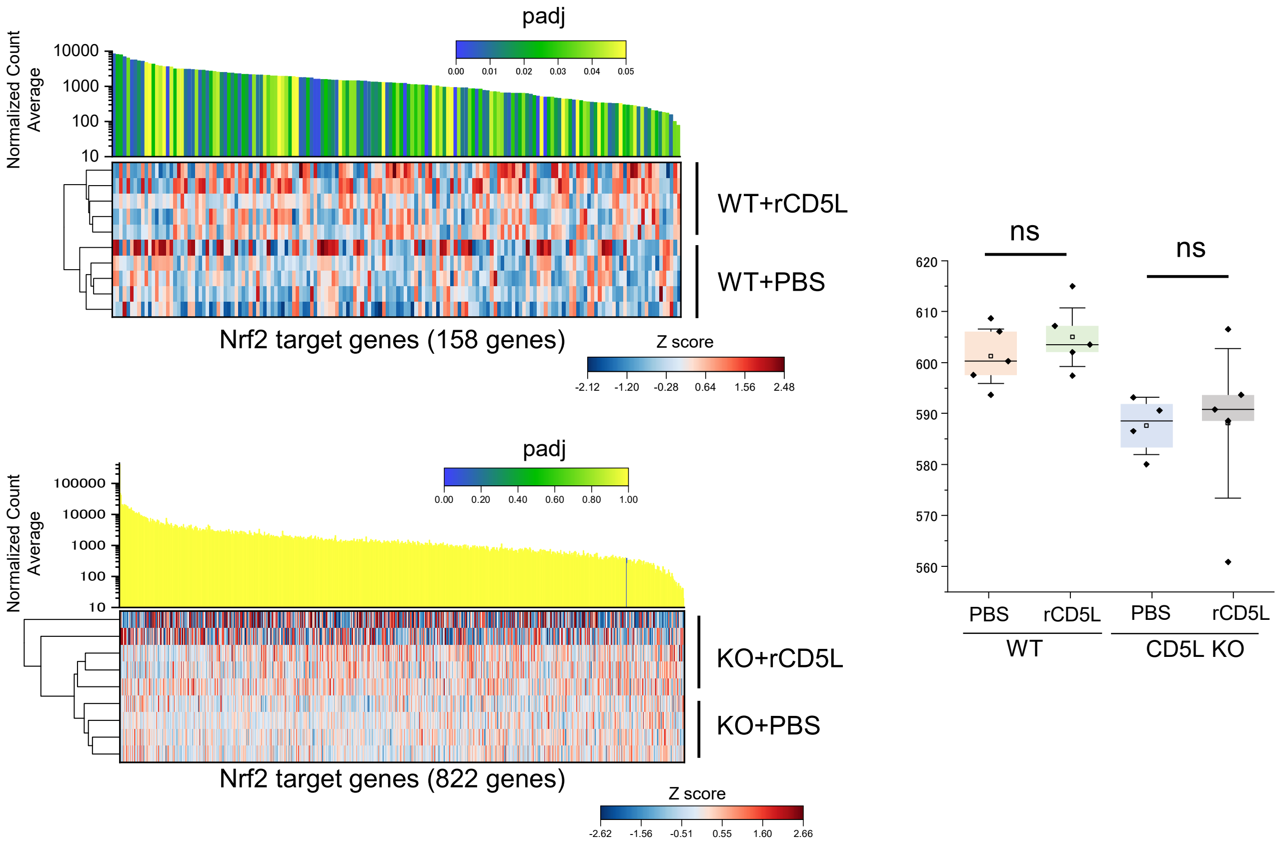

Supplemental Fig. 6. RNAseq analysis of UUO kidney for Nrf2 target genes in WT and CD5L KO mice with or without rCD5L treatment. Expression changes in Nrf2 downstream genes referring to the Nrf2-ome dataset (Türei et al, 2013) in kidney tissues from UUO-treated WT and CD5L KO mice. DEGs were identified within genotype (WT and CD5L KO), contrasting rCD5L vs PBS in each (upper panel: WT+rCD5L vs WT+PBS; lower panel: KO+rCD5L vs KO+PBS). In WT, 158 DEGs (padj < 0.05, Benjamini–Hochberg) were detected and are displayed. In CD5L KO, few genes met this threshold; therefore, the KO heatmap visualizes the predefined gene set used in the main figure (derived from the WT+PBS vs KO+PBS baseline contrast). Genes are sorted by mean normalized counts across samples. The heatmap shows Z-score–normalized expression, and adjacent bar graphs indicate mean expression levels. Bar colors correspond to padj values. Right panel shows the mean FPKM values of all detected Nrf2-target genes (5,043 genes) across groups (WT+PBS, WT+rCD5L, KO+PBS, KO+rCD5L). Each dot represents an individual animal. Statistical significance was assessed by the Mann–Whitney U test.

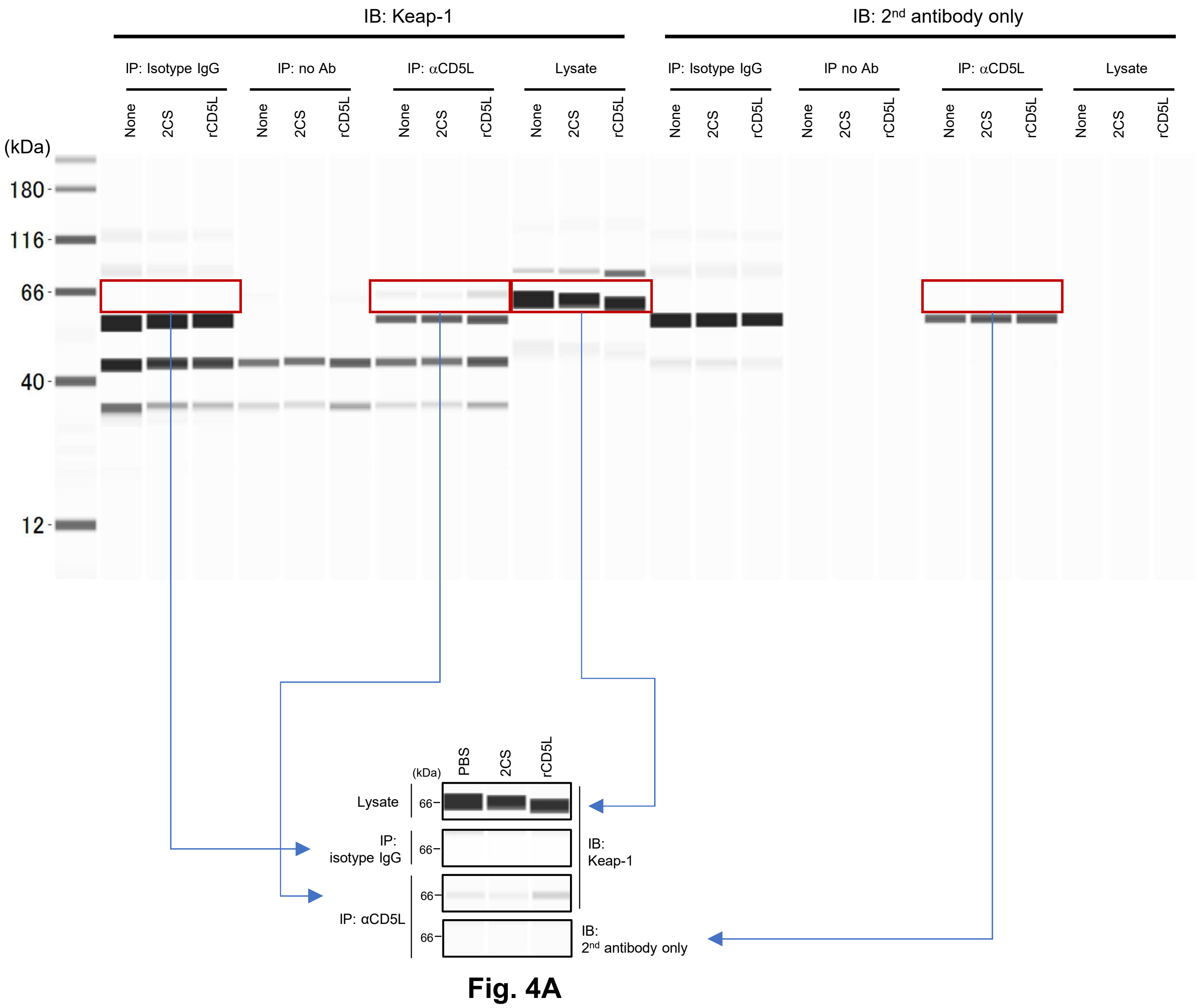

Supplemental Fig. 7A. The whole blot for Keap-1 immunoblotting in co-immunoprecipitation analysis (related to Fig. 4A). rCD5L, 2CS or none was added to the lysates before immunoprecipitation. Detection was performed by WES. The immunoblots for Keap-1 using precipitates ("IP") and the cell lysates are shown. The blots with the 2nd antibody are also presented.

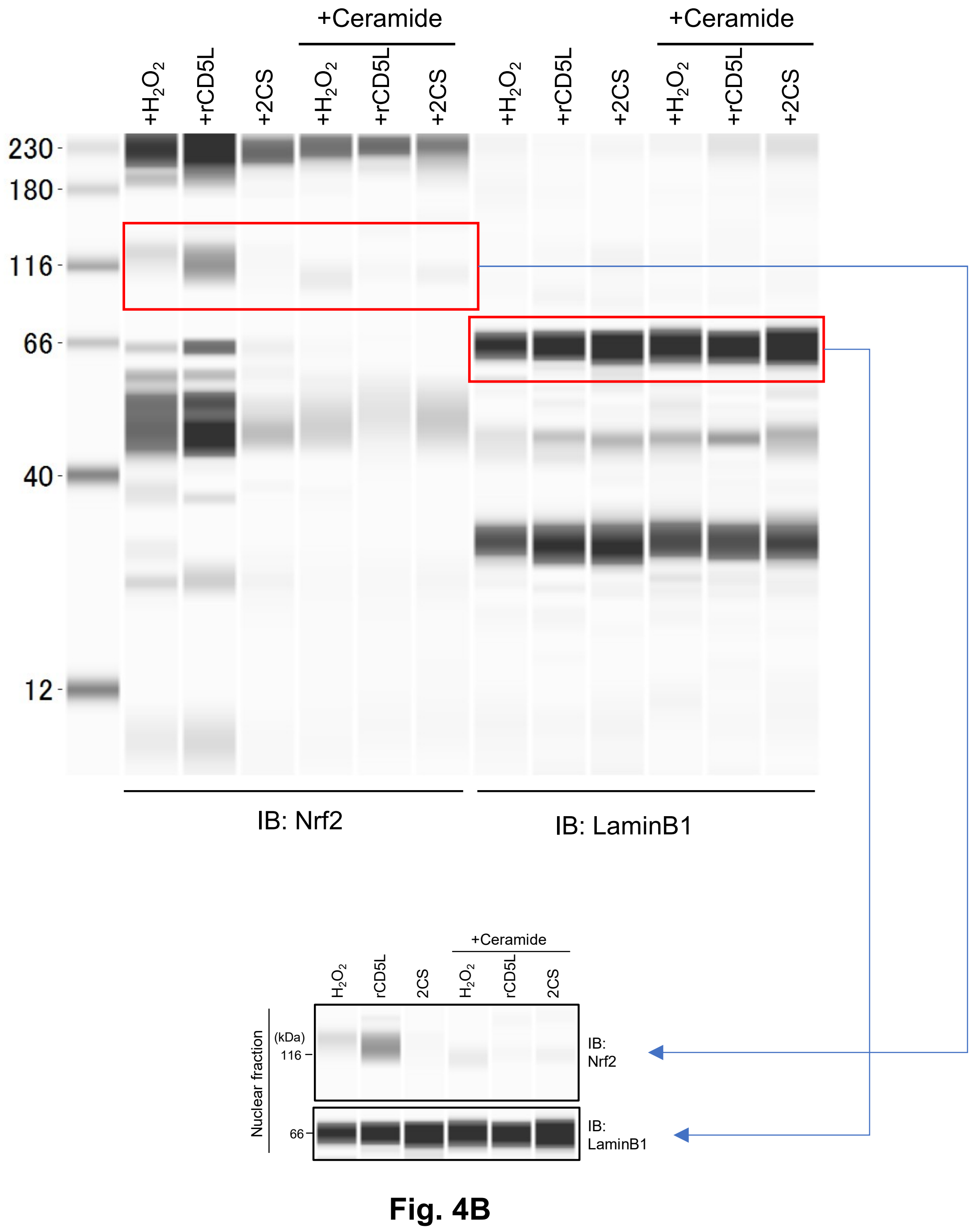

Supplemental Fig. 7B: The whole blot of nuclear NRF2 immunoblotting (related to Fig. 4B). Immunoblotting of Nuclear Nrf2 or laminin B1 from HK-2 cells treated with indicated reagent in the presence or absence of ceramide.

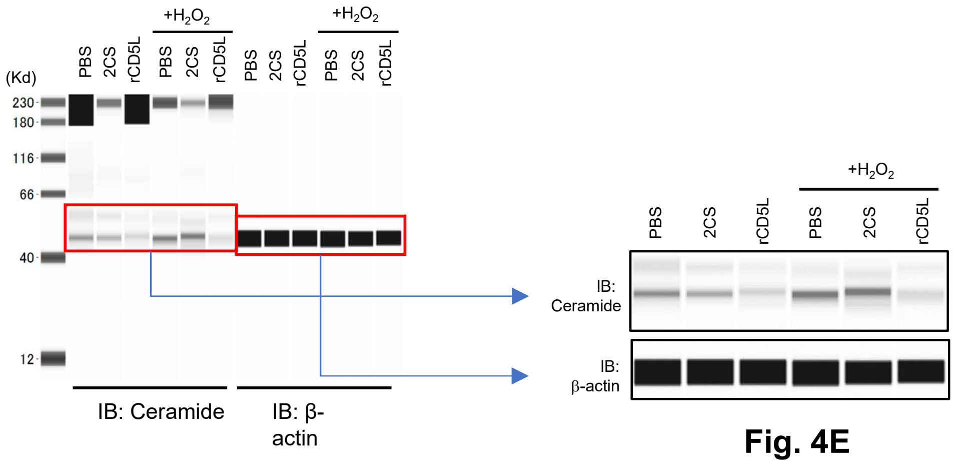

Supplemental Fig. 7C. The whole blot for immunoblotting (WES) for C16 ceramide-bound proteins (related to Fig. 4E). Immunoblotting was performed using lysates of HK-2 cells with or without exposure to H_2O_2 in addition to rCD5L, 2CS or PBS.

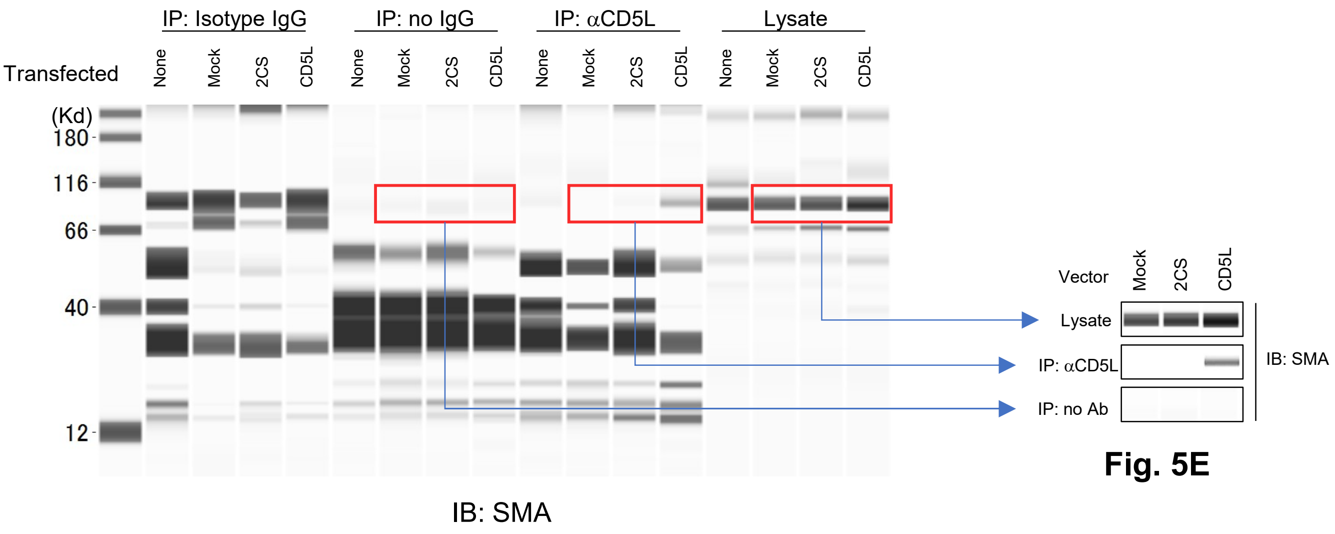

Supplemental Fig. 8A. Whole blots for SMA in a co-immunoprecipitation analysis (related to Fig. 5E). CD5L or 2CS was immunoprecipitated using indicated antibody ("IP") from HK-2 cells transfected with either CD5L, 2CS or Mock vector. The precipitates and the cell lysates were immunoblotted for SMA using WES system.

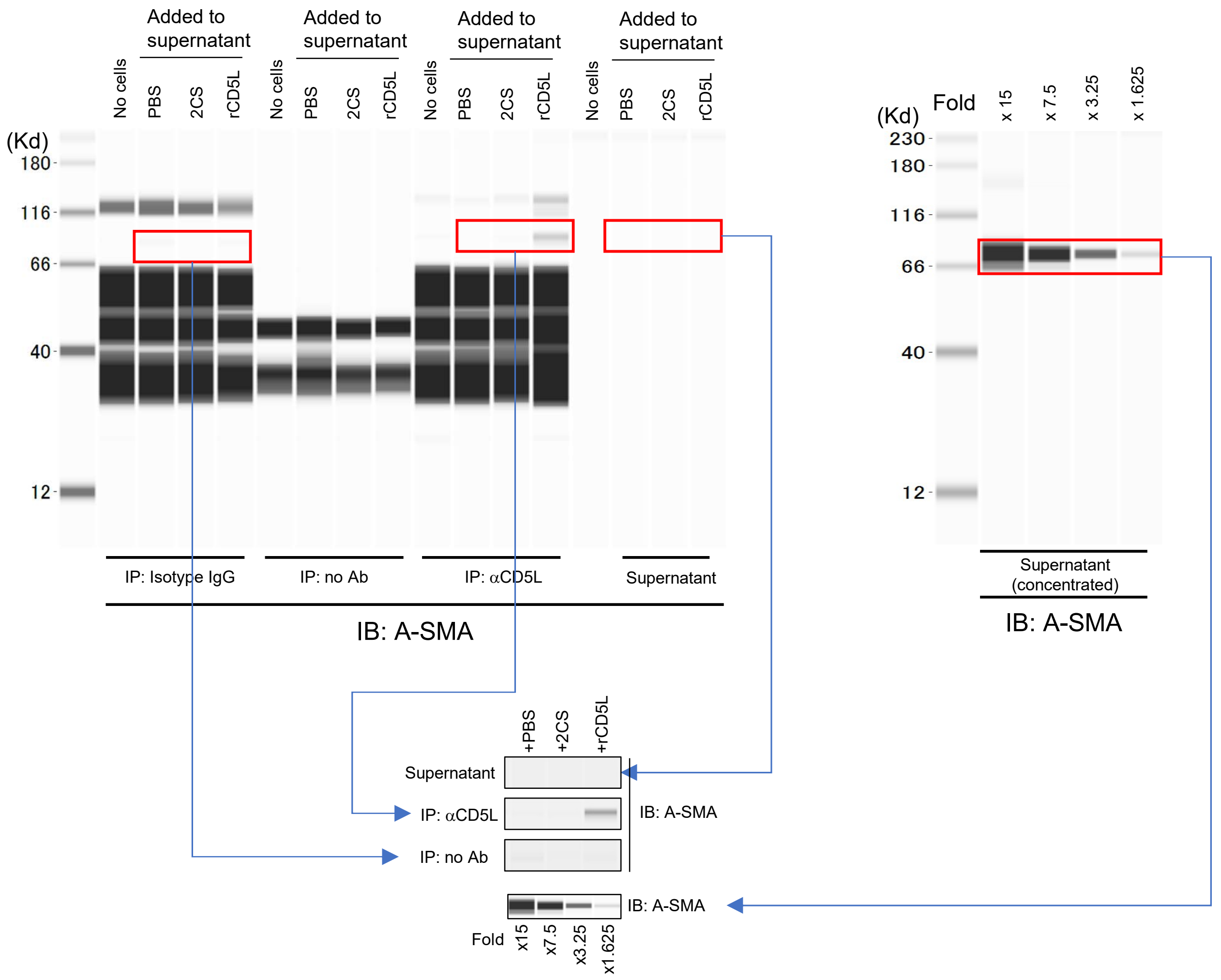

Fig. 5H

Supplemental Fig. 8B: Whole blots for A-SMA immunoblotting in a co-immunoprecipitation analysis (related to Fig. 5H). CD5L or 2CS was immunoprecipitated using indicated antibody ("IP") from the culture supernatant of HK-2 cells added with either PBS, 2CS or rCD5L ("Added to supernatant"), or from the fresh complete culture medium that had not been used for cell culture ("No cells"). The precipitates and the culture supernatants were immunoblotted for SMA using WES system. As the cell culture supernatants contained A-SMA at under detectable levels, we also performed immunoblotting using concentrated supernatants at indicated folds (right panel).
